# Quantum State Assignment Flows

**DOI:** 10.3390/e25091253

**Published:** 2023-08-23

**Authors:** Jonathan Schwarz, Jonas Cassel, Bastian Boll, Martin Gärttner, Peter Albers, Christoph Schnörr

**Affiliations:** 1 Image and Pattern Analysis Group, Institute for Mathematics, Heidelberg University, 69117 Heidelberg, Germany; cassel@math.uni-heidelberg.de (J.C.); bastian.boll@iwr.uni-heidelberg.de (B.B.); schnoerr@math.uni-heidelberg.de (C.S.); 2 Physikalisches Institut, Kirchhoff Institute for Physics, Heidelberg University, 69117 Heidelberg, Germany; martin.gaerttner@kip.uni-heidelberg.de; 3 Research Station Geometry & Dynamics, Institute for Mathematics, Heidelberg University, 69117 Heidelberg, Germany; palbers@mathi.uni-heidelberg.de

**Keywords:** assignment flows, Riemannian gradient flows, density matrix, information geometry, 53B12, 62H35, 68T07

## Abstract

This paper introduces assignment flows for density matrices as state spaces for representation and analysis of data associated with vertices of an underlying weighted graph. Determining an assignment flow by geometric integration of the defining dynamical system causes an interaction of the non-commuting states across the graph, and the assignment of a pure (rank-one) state to each vertex after convergence. Adopting the Riemannian–Bogoliubov–Kubo–Mori metric from information geometry leads to closed-form local expressions that can be computed efficiently and implemented in a fine-grained parallel manner. Restriction to the submanifold of commuting density matrices recovers the assignment flows for categorical probability distributions, which merely assign labels from a finite set to each data point. As shown for these flows in our prior work, the novel class of quantum state assignment flows can also be characterized as Riemannian gradient flows with respect to a non-local, non-convex potential after proper reparameterization and under mild conditions on the underlying weight function. This weight function generates the parameters of the layers of a neural network corresponding to and generated by each step of the geometric integration scheme. Numerical results indicate and illustrate the potential of the novel approach for data representation and analysis, including the representation of correlations of data across the graph by entanglement and tensorization.

## 1. Introduction

### 1.1. Overview and Motivation

A basic task of data analysis is categorization of observed data. We consider the following scenario: On a given undirected, weighted graph G=(V,E,w), data $D_{i}\in X$ are observed as points in a metric space $\left(X,d_{X}\right)$ at each vertex i∈V. Categorization means to determine an assignment

(1) $$D_{i}\;\to \;j\in \left\{1,\cdots ,c\right\}=:\left[c\right]$$

of a *class label j* among a *finite* set of labels to each data point $D_{i}$. Depending on the application, labels carry a specific meaning, e.g., type of tissue in medical imaging data, object type in computer vision or land use in remote sensing data. The decision at any vertex typically depends on decisions at other vertices. Thus, the overall task of labeling data on a graph constitutes a particular form of *structured prediction* in the field of machine learning [1].

*Assignment flows* denote a particular class of approaches for data labeling on graphs [2,3]. The basic idea is to represent each possible label assignment at vertex i∈V by an *assignment vector*$S_{i}\in \Delta_{c}$ in the standard probability simplex, the vertices of which encode the unique label assignment for every label by the corresponding unit vector $e_{j},\;j\in \left[c\right]$. Data labeling is accomplished by computing the flow S(t) of the dynamical system:

(2) $$\dot{S}=R_{S}\left[\Omega S\right],\;S\left(0\right)=S_{0},$$

with the row-stochastic matrix S(t) and row vectors $S_{i}\left(t\right)$ as the state, which, under mild conditions, converges to unique label assignment vectors (unit vectors) at every vertex i∈V [4]. The vector field on the right-hand side in Equation (2) is parameterized by parameters collected in a matrix Ω. These parameters strongly affect the contextual label assignments. They can be learned from data in order to take into account typical relations of data in the current field of application [5]. For a demonstration of the application of this approach to a challenging medical imaging problem, we refer to [6].

From a geometric viewpoint, system (2) can be characterized as a collection of individual flows $S_{i}\left(t\right)$ at each vertex that are *coupled* by the Ω parameters. Each individual flow is determined by a *replicator equation*, which constitutes a basic class of dynamical systems known from evolutionary game theory [7,8]. By restricting each vector $S_{i}\left(t\right)$ to the relative interior ${\overset{˚}{\Delta }}_{c}$ of the probability simplex (i.e., the set of strictly positive discrete probability vectors) and by turning this convex set into a statistical manifold equipped with Fisher–Rao geometry [9], the assignment flow (2) becomes a Riemannian ascent flow on the corresponding product manifold. The underlying information geometry is not only important for making the flow converge to unique label assignments but also for the design of efficient algorithms that actually determine the assignments [10]. For extensions of the basic assignment flow approach to unsupervised scenarios of machine learning and for an in-depth discussion of connections to other closely related work on structured prediction on graphs, we refer to [11,12,13].

In this paper, we study a novel and substantial generalization of assignment flows from a different point of view: assignment of labels to metric data, where the labels are elements of a *continuous* set. This requires replacement of the simplex $\Delta_{c}$ as state space, which can only represent assignments of labels from a *finite* set. The substitutes for assignment vectors $S_{i},\;i\in V$ are Hermitian positive definite *density matrices*$\rho_{i},\;i\in V$ with unit trace:

(3) $$D_{c}=\left\{\rho \in C^{c\times c}:\rho =\rho^{*},\;\mathrm{tr}\rho =1\right\}.$$

Accordingly, the finite set of unit vectors $e_{j},\;j\in \left[c\right]$ (vertices of $\Delta_{c}$ are replaced by *rank-one* density matrices $\rho^{\infty }$, also known as *pure states* in quantum mechanics [14]. The resulting *quantum state assignment flow (QSAF)*

(4) $$\dot{\rho }=R_{\rho }\left[\Omega [\rho ]\right],\;\rho \left(0\right)=\rho_{0},$$

consists of a system of nonlinear first-order differential equations, whose solution ρ(t) evolves on a corresponding product of state spaces $D_{c}$ as given by Equation (3), with a linear averaging operator Ω[.] and a generalized replicator operator $R_{\rho }\left[.\right]$ that is linear with respect to the Ω[ρ] argument and nonlinear with respect to ρ (cf. Equation (64)). The similarity of Equations (4) and (2) can be attributed to the common underlying design strategy. System (4) couples the individual evolutions $\rho_{i}\left(t\right)$ at each vertex i∈V through Ω parameters, and the underlying information geometry causes convergence of each $\rho_{i}\left(t\right)$ towards a pure state. Using a different state space $D_{c}$ (rather than ${\overset{˚}{\Delta }}_{c}$ in Equation (2)) requires the adoption of a different Riemannian metric, which results in a corresponding definition of the operator $R_{\rho }$.

Our approach is natural in that restricting Equation (4) to *diagonal* density matrices results in Equation (2) after identifying each vector $\mathrm{diag}(\rho_{i})$ of diagonal entries of the density matrix $\rho_{i}$ with an assignment vector $S_{i}\in {\overset{˚}{\Delta }}_{c}$. Conversely, Equation (4) considerably generalizes Equation (2) and enhances modeling expressivity due to the *non-commutative* interaction of the state spaces $\rho_{i},\;i\in V$ across the underlying graph G when the quantum state assignment flow is computed by applying geometric numerical integration to Equation (4).

We regard our approach merely as an *approach to data representation and analysis* rather than a contribution to quantum mechanics. For example, dynamics Equation (4) clearly differs from the Hamiltonian evolution of quantum systems, yet we adopt the term “quantum state”, since not only density matrices as state spaces but also the related information geometry are largely motivated by quantum mechanics and quantum information theory [9,15].

### 1.2. Contribution and Organization

Section 2 summarizes the information geometry of both the statistical manifold of categorical distributions and the manifold of strictly positive definite density matrices. Section 3 summarizes the assignment flow approach (2) as a reference for the subsequent generalization to Equation (4). This generalization is the main contribution of this paper and is presented in Section 4. Each row of Table 1 specifies the section where an increasingly general version of the original assignment flow (left column) is generalized to the corresponding quantum state assignment flow (right column, same row).

Alternative metrics on the positive definite matrix manifold that have been used in the literature are reviewed in Section 2.3 in order to position our approach from this point of view. In Section 5, we describe some academic experiments that we conducted to illustrate the properties of the novel approach. Working out a particular scenario of data analysis is beyond the scope of this paper. We conclude and indicate directions of further work in Section 6. For ease of readin, proofs are listed in Appendix A.

This paper considerably elaborates the short preliminary conference version [16].

### 1.3. Basic Notation

For the reader’s convenience, below, we specify the basic notational conventions used in this paper.
[c]           {1,2,⋯,c},c∈N$1_{c}$           ${\left(1,1,\cdots ,1\right)}^{⊤}\in R^{c}$$R_{+}^{c}$           $\left\{x\in R^{c}:x_{i}\geq 0,\;i\in \left[c\right]\right\}$$R_{++}^{c}$           $\left\{x\in R^{c}:x_{i}>0,\;i\in \left[c\right]\right\}$$e_{1},e_{2},\cdots$           Canonical basis vectors of $R^{c}$〈u,v〉           Euclidean inner vector product∥u∥           Euclidean norm $\sqrt{\langle u,u\rangle }$$I_{c}$           Unit matrix of $R^{c\times c}$p·q           Component-wise vector multiplication ${\left(p\cdot q\right)}_{i}=p_{i}q_{i},\;i\in \left[c\right],\;p,q\in R^{c}$$\frac{q}{p}$           Component-wise division ${\left(\frac{q}{p}\right)}_{i}=\frac{q_{i}}{p_{i}},\;i\in \left[c\right],\;q\in R^{c},\;p\in R_{++}^{c}$$H_{c}$           Space of Hermitian c×c matrices (cf. (22))(A)           Trace $\sum_{i}A_{ii}$ of matrix *A*〈A,B〉           Matrix inner product (AB), $A,B\in H_{c}$[A,B]           Commutator AB−BADiag(v)           The diagonal matrix with vector *v* as entriesdiag(V)           The vector of the diagonal entries of a square matrix *V*$\exp_{m}$           The matrix exponential$\log_{m}$           The matrix logarithm $\exp_{m}^{-1}$$\Delta_{c}$           The set of discrete probability vectors of dimension *c* (cf. (6))$S_{c}$           The relative interior of $\Delta_{c}$, i.e., the set of strictly positive probability
vectors (cf. ())$W_{c}$           The product manifold $S_{c}\times \cdots \times S_{c}$ (cf. ())$P_{c}$           The set of symmetric positive definite c×c matrices (cf. (17))$D_{c}$           The subset of matrices in $P_{c}$ whose trace is equal to 1 (cf. (18))$Q_{c}$           The product manifold $D_{c}\times \cdots \times D_{c}$ (cf. (96))$1_{S_{c}}$           Barycenter $\frac{1}{c}1_{c}$ of the manifold $S_{c}$$1_{W_{c}}$           Barycenter ${\left(1_{S_{c}},1_{S_{c}},\cdots ,1_{S_{c}}\right)}^{⊤}$ of the manifold W$1_{D_{c}}$           Matrix $\mathrm{Diag}\left(1_{S_{c}}\right)\in D_{c}\subset C^{c\times c}$$g_{p},g_{W},g_{\rho }$           The Riemannian metrics on $S_{c},W_{c},D_{c}$ (cf. (8), (54), (25))$T_{c,0},T_{c,0},H_{c,0}$           The tangent spaces to $S_{c},W_{c},D_{c}$ (cf. (10), (54), (21))$\pi_{c,0},\Pi_{c,0}$           Orthogonal projections onto $T_{0},H_{c,0}$ (cf. (11), (24))$R_{p},R_{W},R_{\rho }$           Replicator operators associated with the assignment flows
on $S_{c},W_{c},D_{c},Q_{c}$ (cf. (12), (58), (64), (105))*∂*           Euclidean gradient operator: $\partial f\left(p\right)={\left(\partial_{p_{1}}f\left(p\right),\partial_{p_{2}}f\left(p\right),\cdots \right)}^{⊤}$grad           Riemannian gradient operator with respect to the Fisher–Rao metric$R_{W}\left[\cdot \right],\Omega \left[\cdot \right]$, etc.           Square brackets indicate a linear operator that acts in a non-standard
way, e.g., row-wise to a matrix argument.

## 2. Information Geometry

*Information geometry* [17,18] is concerned with the representation of parametric probability distributions from a geometric viewpoint, e.g., the exponential family of distributions [19]. Specifically, an open convex set M of parameters of a probability distribution becomes a Riemannian manifold (M,g) when equipped with a Riemannian metric *g*. The *Fisher–Rao metric* is the canonical choice due to its invariance properties with respect to reparameterization [20]. A closely related scenario concerns the representation of the interior of compact convex bodies as Riemannian manifolds (M,g) due to the correspondence between compactly supported Borel probability measures and an affine equivalence class of convex bodies [21].

A key ingredient of information geometry is the so-called *α-family of affine connections* introduced by Amari [17], which comprises the so-called *e*-connection *∇* and *m*-connection $\nabla^{*}$ as special cases. These connections are torsion-free and dual to each other in the sense that they jointly satisfy the equation that uniquely characterizes the Levi–Civita connection as a metric connection [17] (Definition 3.1, Theorem 3.1). Regarding numerical computations, working with the exponential map induced by the *e* connection is particularly convenient, since its domain is the entire tangent space. We refer to [9,22,23] for further reading and to [24] and [9] (Chapter 7) for the specific case of quantum state spaces.

In this paper, we are concerned with two classes of convex sets:

- The relative interior of probability simplices, each of which represents the categorical (discrete) distributions of the corresponding dimension; and 
- The set of positive definite symmetric matrices with trace one.

Section 2.1 and Section 2.2 introduce the information geometry for the former and the latter class of sets, respectively.

### 2.1. Categorical Distributions

We set

(5) [c]:={1,2,⋯,c},c∈N.

and denote the probability simplex of distributions on [c] as

(6) $$\Delta_{c}:=\left\{p\in R_{+}^{c}:\langle 1_{c},p\rangle =\sum_{i\in [c]}p_{i}=1\right\},\;1_{c}:={\left(1,1,\cdots ,1\right)}^{⊤}\in R^{c}.$$

Its relative interior equipped with the Fisher–Rao metric becomes the Riemannian manifold $\left(S_{c},g\right)$,

(7) $$S_{c}:=\mathrm{rint}\Delta_{c}=\left\{p\in \Delta_{c}:p_{i}>0,\;i\in \left[c\right]\right\},$$

(8) $$g_{p}\left(u,v\right):=\sum_{i\in [c]}\frac{u_{i}v_{i}}{p_{i}}=\langle u,\mathrm{Diag}{\left(p\right)}^{-1}v\rangle ,\;\forall u,v\in T_{c,0},\;p\in S_{c},$$

with the trivial tangent bundle given by

(9) $$TS_{c}≅S_{c}\times T_{c,0}$$

and the tangent space

(10) $$T_{c,0}:=T_{1_{S_{c}}}S_{c}=\left\{v\in R^{c}:\langle 1_{c},v\rangle =0\right\}.$$

The orthogonal projection onto $T_{c,0}$ is denoted by

(11) $$\pi_{c,0}:R^{c}\to T_{c,0},\;\pi_{c,0}v:=v-\frac{1}{c}\langle 1_{c},v\rangle 1_{c}=\left(I_{c}-1_{c}1_{S_{c}}^{⊤}\right)v.$$

The mapping defined next plays a major role in all dynamical systems under consideration in this paper.

**Definition** **1** **(replicator operator).**  *The replicator operator is the linear mapping of the tangent space*

(12) $$R:S_{c}\times T_{c,0}\to T_{c,0},\;R_{p}v:=\left(\mathrm{Diag}\left(p\right)-pp^{⊤}\right)v,\;p\in S_{c},\;v\in T_{c,0}$$

*parameterized by $p\in S_{c}$.*

The name ‘replicator’ is due to the role of this mapping in evolutionary game theory; see Remark 2 on page 2.

**Proposition** **1** **(properties of** $R_{p}$**).** *Mapping (12) satisfies*

(13) $$\begin{array}{cc} R_{p}1_{c} & =0, \end{array}$$

(14) $$\begin{array}{cc} \pi_{c,0}R_{p} & =R_{p}\pi_{c,0}=R_{p},\;\forall p\in S_{c}. \end{array}$$

*Furthermore, let $f:S_{c}\to R$ be a smooth function and $\tilde{f}:U\to R$ be a smooth extension of f to an open neighborhood U of $S_{c}\subset R^{c}$ with $\tilde{f}{|}_{S_{c}}=f$. Then the Riemannian gradient of f with respect to the Fisher–Rao metric (8) is given by*

(15) $$\mathrm{grad}f\left(p\right)=R_{p}\partial \tilde{f}\left(p\right).$$

**Proof.**  Appendix A.1    □

**Remark** **1.** *Equations (15) and (A24) show that the replicator operator $R_{p}$ is the inverse metric tensor with respect to the Fisher–Rao metric (8), as expressed in the ambient coordinates.*

The exponential map induced by the *e* connection is defined on the entire space $T_{c,0}$ and reads [22]

(16) $$\mathrm{Exp}:S_{c}\times T_{c,0}\to S_{c},\;{\mathrm{Exp}}_{p}\left(v\right):=\frac{p\cdot e^{\frac{v}{p}}}{\langle p,e^{\frac{v}{p}}\rangle },\;p\in S_{c},\;v\in T_{c,0}.$$

### 2.2. Density Matrices

We denote the open convex cone of positive definite matrices by

(17) $$P_{c}:=\left\{\rho \in C^{c\times c}:\rho =\rho^{*},\;\rho ≻0\right\}$$

and the manifold of strictly positive definite density matrices by

(18) $$D_{c}:=\left\{\rho \in P_{c}:\mathrm{tr}\rho =1\right\}.$$

where $D_{c}$ is the intersection of $P_{c}$ and the hyperplane defined by the trace-one constraint. Its closure ${\bar{D}}_{c}$ is convex and compact. We can identify the space $D_{c}$ as the space of invertible density operators in the sense of quantum mechanics on the finite-dimensional Hilbert space $C^{c}$ without loss of generality. Any matrix ensemble of the form

(19) $${\left\{M_{i}\right\}}_{i\in [n]}\subset {\bar{P}}_{c}:\;\sum_{i\in [n]}M_{i}=I_{c}$$

induces the probability distribution on [n] via the Born rule

(20) $$p\in \Delta_{n}:\;p_{i}=\langle M_{i},\rho \rangle =\mathrm{tr}(M_{i}\rho ),\;i\in \left[n\right].$$

Equation (19) is called the *positive operator valued measure (POVM)*. We refer to [14] for the physical background and to [25] and references therein for the mathematical background.

The analog of Equation (10) is the tangent space in which at any point, $\rho \in D_{c}$ is equal to the space of traceless symmetric matrices.

(21) $$\begin{array}{cc} H_{c,0}\; & :=H_{c}\cap \left\{X\in C^{c\times c}:\mathrm{tr}X=0\right\}, \end{array}$$

where

(22) $$\begin{array}{cc} H_{c} & :=\{X\in C^{c\times c}:X^{*}=X\}. \end{array}$$

Therefore, the manifold $D_{c}$ has a trivial tangent bundle given by

(23) $$TD_{c}=D_{c}\times H_{c,0},$$

with the tangent space $H_{c,0}=T_{1_{D_{c}}}D_{c}$ defined in Equation (21). The corresponding orthogonal projection onto the tangent space $H_{c,0}$ reads

(24) $$\Pi_{c,0}:H_{c}\to H_{c,0},\;\Pi_{c,0}\left[X\right]:=X-\frac{\mathrm{tr}X}{c}I_{c}.$$

Equipping the manifold $D_{c}$ as defined in Equation (18) with the *Bogoliubov–Kubo–Mori (BKM) metric* [26] results in a Riemannian manifold $\left(D_{c},g\right)$. Using $T_{\rho }D_{c}=H_{c,0}$, this metric can be expressed by

(25) $$g_{\rho }\left(X,Y\right):=\int_{0}^{\infty }\mathrm{tr}\left(X{\left(\rho +\lambda I\right)}^{-1}Y{\left(\rho +\lambda I\right)}^{-1}\right)d\lambda ,\;X,Y\in H_{c,0},\;\rho \in D_{c}.$$

This metric uniquely ensures the existence of a symmetric e-connection *∇* on $D_{c}$ that is mutually dual to its m-connection $\nabla^{*}$ in the sense of information geometry, leading to a *dually flat* structure $\left(g,\nabla ,\nabla^{*}\right)$ [27] and [9] (Theorem 7.1).

The following map and its inverse, defined in terms of the matrix exponential $\exp_{m}$ and its inverse $\log_{m}=\exp_{m}^{-1}$, are convenient.

(26) $$\begin{array}{cc} T:D_{c}\times H_{c} & \to H_{c}, \end{array}$$

(27) $$\begin{array}{cc} T_{\rho }\left[X\right] & :=\frac{d}{dt}\log_{m}\left(\rho +tX\right){|}_{t=0}=\int_{0}^{\infty }{\left(\rho +\lambda I\right)}^{-1}X{\left(\rho +\lambda I\right)}^{-1}d\lambda , \end{array}$$

(28) $$\begin{array}{cc} T_{\rho }^{-1}\left[X\right]\; & =\frac{d}{dt}\exp_{m}\left(H+tX\right){|}_{t=0}=\int_{0}^{1}\rho^{1-\lambda }X\rho^{\lambda }d\lambda ,\;\rho =\exp_{m}\left(H\right). \end{array}$$

The inner product (25) may now be written in the form of

(29) $$g_{\rho }\left(X,Y\right)=\langle T_{\rho }\left[X\right],Y\rangle ,$$

since the trace is invariant with respect to cyclic permutations of a matrix product as an argument. Likewise,

(30) $$\langle \rho ,X\rangle =\left(\rho X\right)=T_{\rho }^{-1}\left[X\right].$$

We also consider two subspaces on the tangent space $T_{\rho }D_{c}$,

(31) $$\begin{array}{cc} T_{\rho }^{u}D_{c}\; & :=\left\{X\in H_{c,0}:\exists \Omega =-\Omega^{*}\;\mathrm{such}\;\mathrm{that}\;X=\left[\Omega ,\rho \right]\right\}, \end{array}$$

(32) $$\begin{array}{cc} T_{\rho }^{c}D_{c}\; & :=\left\{X\in H_{c,0}:\;\left[\rho ,X\right]=0\right\}, \end{array}$$

which yield the decomposition [9]

(33) $$T_{\rho }D_{c}=T_{\rho }^{c}D_{c}⊕T_{\rho }^{u}D_{c}.$$

In Section 4.5, we use this decomposition to recover the assignment flow for categorical distributions from the quantum state assignment flow by restriction to a submanifold of commuting matrices.

### 2.3. Alternative Metrics and Geometries

The positive definite matrix manifold $P_{c}$ (We confine ourselves in this subsection to the case of of real density matrices, as our main references for comparison only deal with real matrix manifolds) has become a tool for data modelling and analysis during the last two decades. Accordingly, a range of Riemannian metrics exist with varying properties. A major subclass is formed by the O(n)-invariant metrics, including the log-Euclidean, affine-invariant, Bures–Wasserstein and Bogoliubov–Kubo–Mori (BKM) metrics. We refer to [28] for a comprehensive recent survey.

This section provides a brief comparison of the *BKM metric* (25) adopted in this paper with two metrics often employed in the literature: the *affine-invariant metric* and the *log-Euclidean metric*, which may be regarded as ‘antipodal points’ in the space of metrics from the geometric and the computational viewpoint, respectively.

#### 2.3.1. Affine-Invariant Metrics

The affine-invariant metric has been derived in various ways, e.g., based on the canonical matrix inner product on the tangent space [29] (Section 6) or as Fisher–Rao metric on the statistical manifold of centered multivariate Gaussian densities [30]. The metric is given by

(34) $$g_{\rho }\left(X,Y\right)=\mathrm{tr}\left(\rho^{-\frac{1}{2}}X\rho^{-\frac{1}{2}}\rho^{-\frac{1}{2}}Y\rho^{-\frac{1}{2}}\right)=\mathrm{tr}\left(\rho^{-1}X\rho^{-1}Y\right),\;\rho \in P_{c},\;X,Y\in T_{\rho }P_{c}.$$

The exponential map with respect to the Levi–Civita connection reads

(35) $$\exp_{\rho }^{\left(\mathrm{aff}\right)}\left(X\right)=\rho^{\frac{1}{2}}\exp_{m}\left(\rho^{-\frac{1}{2}}X\rho^{-\frac{1}{2}}\right)\rho^{\frac{1}{2}},\;\rho \in P_{c},\;X\in T_{\rho }P_{c}.$$

This Riemannian structure turns $P_{c}$ into a manifold with negative sectional curvature [31] (Chapter II.10), which is convenient from the geometric viewpoint due to uniquely defined Riemannian means and geodesic convexity [32] (Section 6.9). On the other hand, evaluating Equations (34) and (35) is computationally expensive, in particular when computing the quantum state assignment flow, which essentially involves geometric averaging.

#### 2.3.2. Log-Euclidean Metric

The log-Euclidean metric introduced in [33] is the pullback of the canonical matrix inner product with respect to the matrix logarithm and is given by

(36) $$g_{\rho }\left(X,Y\right)=\mathrm{tr}\left(d\log_{m}\left(\rho \right)\left[X\right],d\log_{m}\left(\rho \right)\left[Y\right]\right)\overset{\left(27\right)}{=}\langle T_{\rho }\left[X\right],T_{\rho }\left[Y\right]\rangle ,\;\rho \in P_{c}\;X,Y\in T_{\rho }P_{c}.$$

The exponential map reads

(37) $$\exp_{\rho }^{\left(\log\right)}\left(X\right)=\exp_{m}\left(\log_{m}\left(\rho \right)+T_{\rho }\left[X\right]\right),\;\rho \in P_{c}\;X,Y\in T_{\rho }P_{c}$$

and is much more convenient from the computational viewpoint. Endowed with this metric, the space $P_{c}$ is isometric to a Euclidean space. The log-Euclidean metric is not curved and merely invariant under orthogonal transforms and dilations [28].

#### 2.3.3. Comparison to Bogoliubov-Kubo-Mori Metric

The BKM metric (Equations (25) and (30)) given by

(38) $$g_{\rho }\left(X,Y\right)=\langle T_{\rho }\left[X\right],Y\rangle ,\;\rho \in P_{c}\;X,Y\in T_{\rho }P_{c},$$

looks similar to the log-Euclidean metric (36). Regarding them both as members of the class of *mean kernel metrics* [28] (Definition 4.1) enables an intuitive comparison. For real-valued matrices, mean kernel metrics have the form of

(39) $$g_{\rho }\left(X,X\right)=g_{D}\left(X^{\prime },X^{\prime }\right)=\sum_{i,j\in [c]}\frac{{\left(X_{ij}^{\prime }\right)}^{2}}{\phi (D_{ii},D_{jj})},\;\rho =VDV^{⊤},\;V\in O\left(n\right),\;X=VX^{\prime }V^{⊤},\;$$

with a diagonal matrix $D=\mathrm{Diag}(D_{11},\cdots ,D_{cc})$ and a bivariate function $\phi \left(x,y\right)=a\;m{\left(x,y\right)}^{\theta }$, a>0 in terms of a symmetric homogeneous mean $m:R_{+}\times R_{+}\to R_{+}$. Regarding the log-Euclidean metric, $\phi \left(x,y\right)={\left(\frac{x-y}{\log x-\log y}\right)}^{2}$, whereas for the BKM metric, $\phi \left(x,y\right)=\frac{x-y}{\log x-\log y}$.

Taking the restriction to density matrices $D_{c}\subset P_{c}$ into account, one has the relation

(40) $$\begin{array}{cc} \exp_{\rho }^{\left(\log\right)}\left(Y\right)\; & ={\mathrm{Exp}}_{\rho }^{\left(e\right)}\left(X\right),\;\rho \in D_{c},\;X\in H_{c,0}, \end{array}$$

(41) $$\begin{array}{cc} Y & =X-\log\left(\mathrm{tr}\exp_{m}\left(\log_{m}\left(\rho \right)+T_{\rho }\left[X\right]\right)\right)\rho , \end{array}$$

as explained in Remark 4. Here, the left-hand side of Equation (40) is the exponential map (37) induced by the log-Euclidean metric, and ${\mathrm{Exp}}_{\rho }^{\left(e\right)}$ is the exponential map with respect to the affine e connection of information geometry, as detailed below by Proposition 4. This close relationship of the e-exponential map ${\mathrm{Exp}}_{\rho }^{\left(e\right)}$ with the exponential map of the log-Euclidean metric highlights the computational efficiency of using the BKM metric, which we adopt for our approach. This is also motivated by the lack of an explicit formula for the exponential map with respect to the Levi–Civita connection [34]. To date, the sign of the curvature remains unknown.

We note that to the best of our knowledge, the introduction of the affine connections of information geometry as surrogates of the Riemannian connection for any statistical manifold predates the introduction of the log-Euclidean metric for the specific space $P_{c}$.

## 3. Assignment Flows

The assignment flow approach was informally introduced in Section 1. In this section, we summarize the mathematical ingredients of this approach as a reference for the subsequent generalization to quantum states (density matrices) in Section 4. Section 3.1 and Section 3.2 introduce the assignment flow on a single vertex and on an arbitrary graph, respectively. A reparameterization turns the latter into a Riemannian gradient flow (Section 3.3). Throughout this section, we refer to definitions and notions introduced in Section 2.1.

### 3.1. Single-Vertex Assignment Flow

Let $D={\left(D_{1},\cdots ,D_{c}\right)}^{⊤}\in R^{c}$ and consider the task of picking the smallest components of *D*. Formulating this operation as an optimization problem amounts to evaluating the negative support function, in the sense of convex analysis [35] (p. 28), of the probability simplex $\Delta_{c}$ at −D,

(42) $$\min_{j\in [c]}\left\{D_{1},\cdots ,D_{c}\right\}=-\max_{p\in \Delta_{c}}\langle -D,p\rangle .$$

In practice, the vector *D* represents real-valued noisy measurements at some vertex i∈V of an underlying graph G=(V,E) and is therefore in a “general position”, that is, the minimal component is unique. If $j^{*}\in \left[c\right]$ indexes the minimal component $D_{j^{*}}$, then the corresponding unit vector $p^{*}=e_{j^{*}}$ maximizes the right-hand side of (42). *Assignment vectors* assign a label (index) to observed data vectors.

If *D* varies, the operation (42) is non-smooth. In view of a desired interaction of label assignments across the graph (cf. Section 3.2), we therefore replace this operation by a *smooth* dynamical system whose solution converges to the desired assignment vector. To this end, the vector *D* is represented on $S_{c}$ as a *likelihood vector*

(43) $$L_{p}\left(D\right):=\exp_{p}\left(-\pi_{c,0}D\right)\overset{\left(14\right)}{=}\exp_{p}\left(-D\right),\;p\in S_{c},$$

where

(44) $$\exp:S_{c}\times T_{c,0}\to S_{c},\;\exp_{p}\left(v\right):={\mathrm{Exp}}_{p}\circ R_{p}\left(v\right)=\frac{p\cdot e^{v}}{\langle p,e^{v}\rangle },\;p\in S_{c}.$$

The *single-vertex assignment flow* equation reads

(45) $$\dot{p}=R_{p}L_{p}\left(D\right)=p\cdot \left(L_{p}\left(D\right)-\langle p,L_{p}\left(D\right)\rangle 1_{c}\right),\;p\left(0\right)=1_{S_{c}}.$$

Its solution p(t) converges to the vector that solves the label assignment problem (42) (see Corollary 1 below).

**Remark** **2** **(replicator equation).** *Differential equations of the form (45), with some $R^{c}$-valued function F(p) in place of $L_{p}\left(D\right)$, are known as replicator equation in evolutionary game theory [7].*

**Lemma** **1.** *Let $p\in S_{c}$. Then, the differentials of the mapping (44) with respect to p and v are given by*

(46) $$\begin{array}{cc} d_{v}\exp_{p}\left(v\right)\left[u\right]\; & =R_{\exp_{p}\left(v\right)}u, \end{array}$$

(47) $$\begin{array}{cc} d_{p}\exp_{p}\left(v\right)\left[u\right]\; & =R_{\exp_{p}\left(v\right)}\frac{u}{p},\;p\in S_{c},\;u,v\in T_{c,0}. \end{array}$$

**Proof.**  Appendix A.2.    □

**Theorem** **1** **(single-vertex assignment flow).** *The single-vertex assignment flow Equation (45) is equivalent to the system*

(48) $$\begin{array}{cc} \dot{p} & =R_{p}q,\;p\left(0\right)=1_{S_{c}}, \end{array}$$

(49) $$\begin{array}{cc} \dot{q} & =R_{q}q,\;q\left(0\right)=L_{1_{S_{c}}}\left(D\right), \end{array}$$

*with solution given by*

(50) $$\begin{array}{cc} p(t) & =\exp_{1_{S_{c}}}\left(\int_{0}^{t}q\left(\tau \right)d\tau \right). \end{array}$$

**Proof.**  Appendix A.2.    □

**Corollary** **1** **(single-vertex label assignment).** Let $J^{*}:=\arg\min_{j\in [c]}\left\{D_{j}:j\in \left[c\right]\right\}\subseteq \left[c\right]$. Then, the solution p(t) to (45) satisfies

(51) $$\lim_{t\to \infty }p\left(t\right)=\frac{1}{|J^{*}|}\sum_{j\in J^{*}}e_{j}\in \arg\max_{p\in \Delta_{c}}\langle -D,p\rangle .$$

In particular, if D has a unique minimal component $D_{j^{*}}$, then $p\left(t\right)\to e_{j^{*}}$ as t→∞.

**Proof.**  Appendix A.2.    □

### 3.2. Assignment Flows

The assignment flow approach consists of the weighted interaction–as defined below–of single-vertex assignment flows associated with vertices i∈V of a weighted graph G=(V,E,ω) with a non-negative weight function

(52) $$\omega :E\to R_{+},\;ik\mapsto \omega_{ik}.$$

The assignment vectors are denoted by $W_{i},\;i\in V$ and form the row vectors of a row-stochastic matrix.

(53) $$W\in W_{c}:=\underset{|V|\;\mathrm{factors}}{\underset{︸}{S_{c}\times \cdots \times S_{c}}}.$$

The product space $W_{c}$ is called the *assignment manifold*$\left(W_{c},g\right)$, where the metric *g* is defined by applying (8) row-wise,

(54) $$g_{W}\left(U,V\right):=\sum_{i\in V}g_{W_{i}}\left(U_{i},V_{i}\right),\;U,V\in T_{c,0}:=T_{c,0}\times \cdots \times T_{c,0}.$$

The *assignment flow equation* generalizing (45) reads

(55) $$\dot{W}=R_{W}\left[S\left(W\right)\right],$$

where the *similarity vectors*

(56) $$S_{i}\left(W\right):={\mathrm{Exp}}_{W_{i}}\left(\sum_{k\in N_{i}}\omega_{ik}{\mathrm{Exp}}_{W_{i}}^{-1}\left(L_{W_{k}}\left(D_{k}\right)\right)\right),\;i\in V$$

form the row vectors of the matrix $S\left(W\right)\in W_{c}$. The neighborhoods

(57) $$N_{i}:=\left\{i\right\}\cup \left\{k\in V:ik\in E\right\}$$

are defined by the adjacency relation of the underlying graph G, and $R_{W}\left[\cdot \right]$ of Equation (55) applies Equation (12) row-wise,

(58) $$R_{W}{\left[S\left(W\right)\right]}_{i}=R_{W_{i}}S_{i}\left(W\right),\;i\in V.$$

Note that the similarity vectors $S_{i}\left(W\right)$ given by (56) result from geometric weighted averaging of the velocity vectors ${\mathrm{Exp}}_{W_{i}}^{-1}\left(L_{W_{k}}\left(D_{k}\right)\right)$. The velocities represent given data $D_{i},\;i\in V$ via the likelihood vectors $L_{W_{i}}\left(D_{i}\right)$ given by (43). Each choice of the weights $\omega_{ik}$ in (56) associated with every edge ik∈E defines an assignment flow W(t) solving (55). Thus, these weight parameters determine how individual label assignments by (43) and (45) are *regularized*.

Well-posedness, stability and quantitative estimates of basins of attraction to integral label assignment vectors were established in [4]. Reliable and efficient algorithms for numerical computation of the assignment flow were devised in [10].

### 3.3. Reparameterized Assignment Flows

In [36] (Proposition 3.6), the following parameterization of the general assignment flow Equation (55) was introduced, which generalizes the parameterization (48) and (49) of the single-vertex assignment flow (45).

(59) $$\begin{array}{cc} \dot{W} & =R_{W}\left[\bar{S}\right],\;W\left(0\right)=1_{W_{c}}, \end{array}$$

(60) $$\begin{array}{cc} \dot{\bar{S}} & =R_{\bar{S}}\left[\Omega \bar{S}\right],\;\bar{S}\left(0\right)=S\left(1_{W_{c}}\right), \end{array}$$

with the non-negative weight matrix corresponding to the weight function (52),

(61) $$\Omega ={\left(\Omega_{1},\cdots ,\Omega_{\left|V\right|}\right)}^{⊤}\in R^{\left|V|\times |V\right|},\;\;\Omega_{ik}:=\left\{\begin{array}{cc} \omega_{ik}, & \mathrm{if}\;k\in N_{i},\\ 0, & \mathrm{otherwise}. \end{array}\right.$$

In terms of (60), this formulation reveals the “essential” part of the assignment flow equation, since (59) depends on (60) but not vice versa. Furthermore, the data and weights show up only in the initial point and in the vector field on the right-hand side of (60), respectively.

Henceforth, we solely focus on (60) rewritten for convenience as

(62) $$\dot{S}=R_{S}\left[\Omega S\right],\;S\left(0\right)=S_{0},$$

where $S_{0}$ comprises the similarity vectors (56) evaluated at the barycenter $W=1_{W_{c}}$.

## 4. Quantum State Assignment Flows

In this section, we generalize the assignment flow Equations (55) and (62) to the product manifold $Q_{c}$ of density matrices as state space. The resulting equations have a similar mathematical form. Their derivation requires:

- Determination of the form of the Riemannian gradient of functions $f:D_{c}\to R$ with respect to the BKM metric (25), the corresponding replicator operator and exponential mappings Exp and exp, together with their differentials (Section 4.1);
- Definition of the single-vertex quantum state assignment flow (Section 4.2);
- Determination of the general quantum state assignment flow equation for an arbitrary graph (Section 4.3) and its alternative parameterization (Section 4.4), which generalizes Formulation (62) of the assignment flow accordingly.

A natural question is: What does “label” mean for a generalized assignment flow evolving on the product manifold $Q_{c}$ of density matrices? For the single vertex quantum state assignment flow, i.e., without interaction of these flows on a graph, it turns out that the pure state corresponding to the minimal eigenvalue of the initial density matrix is assigned to the given data point (Proposition 5). Coupling non-commuting density matrices over the graph through the novel quantum state assignment flow, therefore, generates interesting complex dynamics, as illustrated in Section 5. In Section 4.5, we show that the restriction of the novel quantum state assignment flow to commuting density matrices recovers the original assignment flow for discrete labels.

Throughout this section, we refer to definitions and notions introduced in Section 2.2.

### 4.1. Riemannian Gradient, Replicator Operator and Further Mappings

**Proposition** **2** **(Riemannian gradient).** *Let $f:D_{c}\to R$ be a smooth function defined on the manifold (18) and $\tilde{f}:U\to R$ be a smooth extension of f to an open neighborhood U of $D_{c}\subset C^{c\times c}$ with $\tilde{f}{|}_{D_{c}}=f$. Then, its Riemannian gradient with respect to the BKM metric (25) is given by*

(63) $${\mathrm{grad}}_{\rho }f=T_{\rho }^{-1}\left[\partial \tilde{f}\right]-\langle \rho ,\partial \tilde{f}\rangle \rho ,$$

*where $T_{\rho }^{-1}$ is given by (28), and $\partial \tilde{f}$ is the ordinary gradient with respect to the Euclidean structure of the ambient space $C^{c\times c}$.*

**Proof.**  Appendix A.3.    □

Comparing the result (63) with (15) motivates the following:

(64) $$R_{\rho }:H_{c}\to H_{c,0},\;R_{\rho }\left[X\right]:=T_{\rho }^{-1}\left[X\right]-\langle \rho ,X\rangle \rho ,\;\rho \in D_{c}\;\left(\mathrm{replicator}\mathrm{map}\right)$$

The following lemma shows that the properties (63) extend to (64).

**Lemma** **2** **(properties of** $R_{\rho }$**).** *Let $\Pi_{c,0}$ denote the orthogonal projection (24). Then, the replicator map (64) satisfies*

(65) $$\Pi_{c,0}\circ R_{\rho }=R_{\rho }\circ \Pi_{c,0}=R_{\rho },\;\forall \rho \in D_{c}.$$

**Proof.**  Appendix A.3.    □

Lemma 2 shows that the replicator map (64) implicitly comprises the orthogonal projection onto the tangent space. This allows for the averaging in (109) without the necessity of explicit projection, which simplifies the notation and explains the larger domain $H_{c}$ of $R_{\rho }$ in (64).

Next, using the tangent space $H_{c,0}$, we define a parameterization of the manifold $D_{c}$ in terms of the mapping.

(66) $$\begin{array}{cc} \Gamma :H_{c,0}\to D_{c},\;\Gamma \left(X\right)\; & :=\frac{\exp_{m}\left(X\right)}{\mathrm{tr}\exp_{m}\left(X\right)}=\exp_{m}\left(X-\psi (X)I\right),\;\left(\Gamma -\mathrm{map}\right) \end{array}$$

where

(67) $$\begin{array}{cc} \psi (X) & :=\log(\mathrm{tr}\exp_{m}\left(X\right)). \end{array}$$

The following lemma and proposition show that the domain of Γ extends to $R^{c\times c}$.

**Lemma** **3** (**extension of** Γ). *The extension to $C^{c\times c}$ of the mapping* Γ *defined by (66) is well-defined and given by*

(68) $$\Gamma :C^{c\times c}\to D_{c},\;\Gamma \left(Z\right)=\Gamma \left(\Pi_{c,0}\left[Z\right]\right).$$

**Proof.**  Appendix A.3.    □

**Proposition** **3** (**inverse of** Γ). *The map* Γ *defined by (66) is bijective with inverse*

(69) $$\Gamma^{-1}:D_{c}\to H_{c,0},\;\Gamma^{-1}\left(\rho \right)=\Pi_{c,0}\left[\log_{m}\rho \right].$$

**Proof.**  Appendix A.3.    □

The following lemma provides the differentials of the mappings Γ and $\Gamma^{-1}$.

**Lemma** **4** (**differentials** dΓ **and** $d\Gamma^{-1}$). *Let* $H,X\in H_{c,0}$ *with* Γ(H)=ρ *and* $Y\in TH_{c,0}≅H_{c,0}$*. Then,*

(70) $$\begin{array}{cc} d\Gamma (H)[Y]\; & =T_{\rho }^{-1}\left[Y-\langle \rho ,Y\rangle I\right],\;\rho =\Gamma \left(H\right), \end{array}$$

(71) $$\begin{array}{cc} d\Gamma^{-1}\left(\rho \right)\left[X\right]\; & =\Pi_{c,0}\circ T_{\rho }\left[X\right]. \end{array}$$

**Proof.**  Appendix A.3.    □

We finally compute a closed-form expression of the e-geodesic, i.e., the geodesic resp. exponential map induced by the e connection on the manifold $\left(D_{c},g\right)$.

**Proposition** **4** (**e-geodesics**). *The e-geodesic emanating at $\rho \in D_{c}$ in the direction of $X\in H_{c,0}$ and the corresponding exponential map are given by*

(72) $$\begin{array}{cccc} \gamma_{\rho ,X}^{\left(e\right)}\left(t\right)\; & :={\mathrm{Exp}}_{\rho }^{\left(e\right)}\left(tX\right),\;t\geq 0\; & \; & \left(e-\mathrm{geodesic}\right) \end{array}$$

(73) $$\begin{array}{cccc} {\mathrm{Exp}}_{\rho }^{\left(e\right)}\left(X\right)\; & :=\Gamma \left(\Gamma^{-1}\left(\rho \right)+d\Gamma^{-1}\left(\rho \right)\left[X\right]\right)\; & \; & \left(\mathrm{exponential}\mathrm{map}\right) \end{array}$$

(74) $$\begin{array}{cc} \; & =\Gamma \left(\Gamma^{-1}\left(\rho \right)+\Pi_{c,0}\circ T_{\rho }\left[X\right]\right). \end{array}$$

**Proof.**  Appendix A.3.    □

**Corollary** **2** (**inverse exponential map**). *The inverse of the exponential mapping (72) is given by*

(75) $${\left({\mathrm{Exp}}_{\rho }^{\left(e\right)}\right)}^{-1}:D_{c}\to H_{c,0},\;{\left({\mathrm{Exp}}_{\rho }^{\left(e\right)}\right)}^{-1}\left(\mu \right)=d\Gamma \left(\Gamma^{-1}\left(\rho \right)\right)\left[\Gamma^{-1}\left(\mu \right)-\Gamma^{-1}\left(\rho \right)\right].$$

**Proof.**  Appendix A.3.    □

Analogous to (44), we define the mapping as $\exp_{\rho }$, where both the subscript and the argument disambiguate the meaning of “exp”.

**Lemma** **5** (**exp-map**). *The mapping defined using (73) and (64) by*

(76) $$\begin{array}{cccc} \exp_{\rho }:H_{0,c}\to D_{c},\;\exp_{\rho }\left(X\right)\; & :={\mathrm{Exp}}_{\rho }^{\left(e\right)}\circ R_{\rho }\left[X\right],\;\rho \in D_{c}\; & \; & \left(\exp-\mathrm{map}\right) \end{array}$$

*has the explicit form*

(77) $$\begin{array}{cc} \exp_{\rho }\left(X\right)\; & =\Gamma \left(\Gamma^{-1}\left(\rho \right)+X\right). \end{array}$$

**Proof.**  Appendix A.3.    □

The following lemma provides the explicit form of the differential of the mapping (76} and (77), which resembles the corresponding Formula (46) of the assignment flow.

**Lemma** **6** (**differential $d\exp_{\rho }$**). *The differential of the mapping (76) reads with $\rho \in D_{c}$, $X\in H_{c,0}$ and $Y\in TH_{c,0}≅H_{c,0}$*

(78) $$d\exp_{\rho }\left(X\right)\left[Y\right]=R_{\exp_{\rho }\left(X\right)}\left[Y\right].$$

**Proof.**  Appendix A.3.    □

**Remark** **3** (**comparing exp-maps–I**). *Since (78) resembles (46), one may wonder about the connection between (77) and (44). In view of (66), we define*

(79) $$\gamma :T_{c,0}\to S_{c},\;\gamma \left(v\right):=\frac{e^{v}}{\langle 1,e^{v}\rangle }=\exp_{1_{S_{c}}}\left(v\right)$$

*and compute with the expression for its inverse (cf. [36])*

(80) $$\begin{array}{cc} \gamma^{-1}\left(p\right)\; & =\pi_{c,0}\log\frac{p}{1_{S_{c}}}=\pi_{c,0}\left(\log p-\log1_{S_{c}}\right)=\pi_{c,0}\log p \end{array}$$

(81) $$\begin{array}{cc} \; & \overset{\left(11\right)}{=}\log p-\langle 1_{S_{c}},\log p\rangle 1_{c} \end{array}$$

*which resembles (69). Moreover, in view of (77), the analogous expression using γ instead of* Γ *reads*

(82) $$\begin{array}{cc} \gamma \left(\gamma^{-1}\left(p\right)+v\right)\; & =\frac{e^{\pi_{c,0}\log p+v}}{\langle 1,e^{\pi_{c,0}\log p+v}\rangle }=\frac{\langle 1_{S_{c}},\log p\rangle p\cdot e^{v}}{\langle \langle 1_{S_{c}},\log p\rangle p,e^{v}\rangle }=\frac{p\cdot e^{v}}{\langle p,e^{v}\rangle } \end{array}$$

(83) $$\begin{array}{cc} \; & =\exp_{p}\left(v\right). \end{array}$$

**Remark** **4** (**comparing exp-maps–II**). *Using the above definitions and relations, we check Equation (40): $\exp_{\rho }^{\left(\log\right)}\left(Y\right)={\mathrm{Exp}}_{\rho }^{\left(e\right)}\left(X\right)$, where the relation (41) between Y and X can now be written in the following form:*

(84) $$Y\overset{\left(67\right)}{=}X-\psi \left(\log_{m}\left(\rho \right)+T_{\rho }\left[X\right]\right)\rho .$$

*Direct computation yields*

(85) $$\begin{array}{cc} \exp_{\rho }^{\left(\log\right)}\left(Y\right)\; & \overset{\left(37\right)}{=}\exp_{m}\left(\log_{m}\left(\rho \right)+T_{\rho }\left[Y\right]\right) \end{array}$$

(86) $$\begin{array}{cc} \; & \overset{\left(41\right)}{=}\exp_{m}\left(\log_{m}\left(\rho \right)+T_{\rho }\left[X\right]-\psi \left(\log_{m}\left(\rho \right)+T_{\rho }\left[X\right]\right)\overset{=I_{c}}{\overset{︷}{T_{\rho }\circ \underset{=\rho }{\underset{︸}{T_{\rho }^{-1}\left[I_{c}\right]}}}}\right) \end{array}$$

(87) $$\begin{array}{cc} \; & \overset{\begin{array}{c} \left(66\right)\\ \left(68\right) \end{array}}{=}\Gamma \left(\Pi_{c,0}\left[\log_{m}\left(\rho \right)\right]+\Pi_{c,0}\circ T_{\rho }\left[X\right]\right)=\Gamma \left(\Gamma^{-1}\left(\rho \right)+\Pi_{c,0}\circ T_{\rho }\left[X\right]\right) \end{array}$$

(88) $$\begin{array}{cc} \; & ={\mathrm{Exp}}_{\rho }^{\left(e\right)}\left(X\right). \end{array}$$

### 4.2. Single-Vertex Density Matrix Assignment Flow

We generalize the single-vertex assignment flow Equation (45) to the manifold $\left(D_{c},g_{\rho }\right)$ given by (18) with the BKM metric (25).

In view of (43), the *likelihood matrix* is defined as

(89) $$L_{\rho }:H_{c}\to D_{c},\;L_{\rho }\left(D\right):=\exp_{\rho }\left(-\Pi_{c,0}\left[D\right]\right),\;\rho \in D_{c},$$

and the corresponding *single-vertex quantum state assignment flow (SQSAF)* equation reads

(90) $$\begin{array}{cccc} \dot{\rho } & =R_{\rho }\left[L_{\rho }\left(D\right)\right]\; & \; & \left(\mathrm{SQSAF}\right) \end{array}$$

(91) $$\begin{array}{cc} \; & \overset{\left(64\right)}{=}T_{\rho }^{-1}\left[L_{\rho }\left(D\right)\right]-\langle \rho ,L_{\rho }\left(D\right)\rangle \rho ,\;\rho \left(0\right)=1_{D_{c}}=\mathrm{Diag}\left(1_{S_{c}}\right). \end{array}$$

Proposition 5 below specifies its properties after a preparatory Lemma.

**Lemma** **7.** *Assume*

(92) $$D=Q\Lambda_{D}Q^{⊤}\in H_{c}\;\mathrm{and}\;\rho =Q\Lambda_{\rho }Q^{⊤}\in D_{c}$$

*can be simultaneously diagonalized with Q∈O(c), $\Lambda_{D}=\mathrm{Diag}\left(\lambda_{D}\right)$, $\Lambda_{\rho }=\mathrm{Diag}\left(\lambda_{\rho }\right)$ and $\lambda_{\rho }\in S_{c}$, since trρ=1. Then,*

(93) $$L_{\rho }\left(D\right)=Q\mathrm{Diag}\left(\exp_{\lambda_{\rho }}\left(-\lambda_{D}\right)\right)Q^{⊤}.$$

**Proof.**  Appendix A.3.    □

**Proposition** **5** (**SQSAF limit**). *Let $D=Q\Lambda_{D}Q^{⊤}$ be the spectral decomposition of D with eigenvalues $\lambda_{1}\geq \cdots \geq \lambda_{c}$ and orthonormal eigenvectors of $Q=(q_{1},\cdots ,q_{c})$. Assume that the minimal eigenvalue $\lambda_{c}$ is unique. Then, the solution ρ(t) to (90) satisfies*

(94) $$\lim_{t\to \infty }\rho \left(t\right)=\Pi_{q_{c}}:=q_{c}q_{c}^{⊤}.$$

**Proof.**  Appendix A.3.    □

### 4.3. Quantum State Assignment Flow

This section describes our main result, i.e,. the definition of a novel flow of coupled density matrices in terms of a parameterized interaction of single-vertex flows of the form (90) on a given graph G=(V,E,ω).

We assume the weight function $\omega :E\to R_{+}$ to be non-negative with $\omega_{ij}=0$ if ij∉E and

(95) $$\sum_{k\in N_{i}}\omega_{ik}=1,$$

where we adopt the notation (57) for neighborhoods $N_{i},\;i\in V$. Analogous to (53), we define the product manifold as

(96) $$\rho \in Q_{c}:=\underset{|V|\;\mathrm{factors}}{\underset{︸}{D_{c}\times \cdots \times D_{c}}}$$

where $D_{c}$ is given by (18). The corresponding factors of ρ are denoted by

(97) $$\rho ={\left(\rho_{i}\right)}_{i\in [c]},\;\rho_{i}\in D_{c},\;i\in V.$$

where $Q_{c}$ becomes a Riemannian manifold when equipped with the the following metric:

(98) $$g_{\rho }\left(X,Y\right):=\sum_{i\in V}g_{\rho_{i}}\left(X_{i},Y_{i}\right),\;X,Y\in TQ_{c}:=H_{c,0}\times \cdots \times H_{c,0},$$

with $g_{\rho_{i}}$ given by (25) for each i∈V. We set

(99) $$1_{Q_{c}}:={\left(1_{D_{c}}\right)}_{i\in V}\in Q_{c},$$

with $1_{D_{c}}$ given by (91). Our next step is to define a *similarity mapping* analogous to (56),

(100) $$S:V\times Q_{c},\;S_{i}\left(\rho \right):={\mathrm{Exp}}_{\rho_{i}}^{\left(e\right)}\left(\sum_{k\in N_{i}}\omega_{ik}{\left({\mathrm{Exp}}_{\rho_{i}}^{\left(e\right)}\right)}^{-1}\left(L_{\rho_{k}}\left(D_{k}\right)\right)\right),$$

based on mappings (73) and (89). Thanks to the use of the exponential map of the e connection, the matrix $S_{i}\left(\rho \right)$ can be rewritten and computed in a simpler, more explicit form.

**Lemma** **8** (**similarity map**). *Equation (100) is equivalent to*

(101) $$S_{i}\left(\rho \right)=\Gamma \left(\sum_{k\in N_{i}}\omega_{ik}\left(\log_{m}\rho_{k}-D_{k}\right)\right).$$

**Proof.**  Appendix A.3.    □

Expression (100), which defines the similarity map, looks like a single iterative step for computing the Riemannian center of mass of the likelihood matrices $\left\{L_{\rho_{k}}\left(D_{k}\right):k\in N_{i}\right\}$ if(!) the exponential map of the Riemannian (Levi Civita) connection were used. Instead, when using the exponential map ${\mathrm{Exp}}^{\left(e\right)}$, $S_{i}\left(\rho \right)$ may be interpreted as carrying out a single iterative step for the corresponding *geometric mean* on the manifold $D_{c}$.

Therefore, a natural idea is to define the similarity map to be this geometric mean rather than just by a single iterative step. Surprisingly, analogous to the similarity map (56) for categorical distributions (cf. [3]), both definitions are *identical*, as shown next.

**Proposition** **6** (**geometric mean property**). *Assume that $\bar{\rho }\in D_{c}$ solves the equation*

(102) $$0=\sum_{k\in N_{i}}\omega_{ik}{\left({\mathrm{Exp}}_{\bar{\rho }}^{\left(e\right)}\right)}^{-1}\left(L_{\rho_{k}}\left(D_{k}\right)\right)$$

*which corresponds to the optimality condition for Riemannian centers of mass [32] (Lemma 6.9.4), except using a different exponential map. Then,*

(103) $$\bar{\rho }=S_{i}\left(\rho \right)$$

*with the right-hand side given by (100).*

**Proof.**  Appendix A.3.    □

We are now in the position to define the *quantum state assignment flow* along the lines of the original assignment flow (55),

(104) $$\dot{\rho }=R_{\rho }\left[S\left(\rho \right)\right],\;\rho \left(0\right)=1_{Q_{c}},\;\;\left(\mathrm{QSAF}\right)$$

where both the replicator map $R_{\rho }$ and the similarity map S(·) apply factor-wise,

(105) $$\begin{array}{cc} S{\left(\rho \right)}_{i} & =S_{i}\left(\rho \right), \end{array}$$

(106) $$\begin{array}{cc} R_{\rho }{\left[S\left(\rho \right)\right]}_{i} & =R_{\rho_{i}}\left[S_{i}\left(\rho \right)\right],\;i\in V \end{array}$$

with the mappings $S_{i}$ given by (101) and $R_{\rho_{i}}$ given by (64).

### 4.4. Reparameterization and Riemannian Gradient Flow

The reparameterization of the assignment flow (59) and (60) for categorical distributions described in Section 3.3 has proven to be useful for characterizing and analyzing assignment flows. Under suitable conditions on the parameter matrix Ω, the flow performs a Riemannian descent flow with respect to a non-convex potential [36] (Proposition 3.9) and has convenient stability and convergence properties [4].

In this section, we derive a similar reparameterization of the quantum state assignment flow (104).

**Proposition** **7** (**reparametrization**). *Define the linear mapping as*

(107) $$\Omega :Q_{c}\to Q_{c},\;\Omega {\left[\rho \right]}_{i}:=\sum_{k\in N_{i}}\omega_{ik}\rho_{k}.$$

*Then, the density matrix assignment flow equation (104) is equivalent to the following system:*

(108) $$\begin{array}{cc} \dot{\rho } & =R_{\rho }\left[\mu \right],\;\;\;\;\;\rho \left(0\right)=1_{Q_{c}}, \end{array}$$

(109) $$\begin{array}{cc} \dot{\mu } & =R_{\mu }\left[\Omega [\mu ]\right],\;\mu \left(0\right)=S\left(1_{Q_{c}}\right). \end{array}$$

**Proof.**  Appendix A.3.    □

For the following, we adopt the *symmetry assumption.*

(110) $$\begin{array}{c} \omega_{ij}=\omega_{ji},\;\forall i,j\in V \end{array}$$

(111) $$\begin{array}{c} j\in N_{i}\;\Leftrightarrow \;i\in N_{j},\;i,j\in V. \end{array}$$

As a consequence, the mapping (107) is self-adjoint.

(112) $$\begin{array}{cc} \langle \mu ,\Omega [\rho ]\rangle \; & =\sum_{i\in V}\langle \mu_{i},\Omega {\left[\rho \right]}_{i}\rangle =\sum_{i\in V}\sum_{k\in N_{i}}\omega_{ik}\langle \mu_{i},\rho_{k}\rangle =\sum_{i\in V}\sum_{k\in N_{i}}\omega_{ki}\langle \mu_{i},\rho_{k}\rangle \end{array}$$

(113) $$\begin{array}{cc} \; & =\sum_{k\in V}\sum_{i\in N_{k}}\omega_{ki}\langle \mu_{i},\rho_{k}\rangle =\sum_{k\in N_{i}}\langle \Omega {\left[\mu \right]}_{k},\rho_{k}\rangle =\langle \Omega \left[\mu \right],\rho \rangle . \end{array}$$

**Proposition** **8** (**Riemannian gradient QSAF flow**). *Suppose that the mapping Ω[·] given by (107) is self-adjoint with respect to the canonical matrix inner product. Then, the solution μ(t) to (109) also solves*

(114) $$\begin{array}{cc} \dot{\mu }\; & =-{\mathrm{grad}}_{\mu }J\left(\mu \right)\;\mathrm{with}\;{\left({\mathrm{grad}}_{\mu }J\left(\mu \right)\right)}_{i}={\mathrm{grad}}_{\mu_{i}}J\left(\mu \right) \end{array}$$

*with respect to the potential*

(115) $$\begin{array}{cc} J(\mu ) & :=-\frac{1}{2}\langle \mu ,\Omega \left[\mu \right]\rangle . \end{array}$$

**Proof.**  Appendix A.3.    □

We conclude this section by rewriting the potential in a more explicit, informative form.

**Proposition** **9** (**nonconvex potential**). *We define*

(116) $$L_{G}:Q_{c}\to Q_{c},\;L_{G}:=\mathrm{id}-\Omega$$

*with* Ω *given by (107). Then, the potential (115) can be rewritten as*

(117) $$\begin{array}{cc} J(\mu ) & =\frac{1}{2}\left(\langle \mu ,L_{G}\left[\mu \right]\rangle -{∥\mu ∥}^{2}\right) \end{array}$$

(118) $$\begin{array}{cc} \; & =\frac{1}{4}\sum_{i\in V}\sum_{j\in N_{i}}\omega_{ij}∥\mu_{i}-\mu_{j}{∥}^{2}-\frac{1}{2}{∥\mu ∥}^{2}. \end{array}$$

**Proof.**  Appendix A.3.    □

### 4.5. Recovering the Assignment Flow for Categorical Distributions

In the following, we show how the assignment flow (62) for categorical distributions arises as special case of the quantum state assignment flow under suitable conditions, as detailed below.

**Definition** **2** (**commutative submanifold**). *Let*

(119) $$\Pi =\{\pi_{i}:i\in \left[l\right]\},\;l\leq c$$

*denote a set of operators that orthogonally project onto disjoint subspaces of $C^{c}$,*

(120) $$\begin{array}{cc} \pi_{i}^{2} & =\pi_{i},\;\forall i\in \left[l\right], \end{array}$$

(121) $$\begin{array}{cc} \pi_{i}\pi_{j} & =0,\;\forall i,j\in [l],\;i\neq j, \end{array}$$

*and which are complete in the sense that*

(122) $$\sum_{i\in [l]}\pi_{i}=I_{c}.$$

*Given a family* Π *of operators, we define*

(123) $$D_{\Pi }:=\left\{\sum_{i\in [l]}\frac{p_{i}}{\mathrm{tr}\pi_{i}}\pi_{i}:p\in S_{l}\right\}\subset D_{c}$$

*the submanifold of commuting Hermitian matrices, which can be diagonalized simultaneously.*

A typical example for a family (119) is

(124) $$\Pi_{U}=\left\{\pi_{i}=u_{i}u_{i}^{*}:i\in \left[c\right]\right\},$$

where $U=\{u_{1},\cdots ,u_{c}\}$ is an orthonormal basis of $C^{c}$. The following lemma elaborates the bijection $D_{\Pi }↔S_{l}$.

**Lemma** **9** (**properties of** $D_{\Pi }$). *Let $D_{\Pi }\subset D_{c}$ be given by (123) and denote the corresponding inclusion map by $\iota :D_{\Pi }↪D_{c}$. Then,*

*(a)*  The submanifold $\left(D_{\Pi },\iota^{*}g_{\mathrm{BKM}}\right)$ with the induced BKM metric is isometric to $\left(S_{l},g_{\mathrm{FR}}\right)$;
*(b)*  *If $\mu \in D_{\Pi }$, then the tangent subspace $T_{\mu }D_{\Pi }$ is contained in the subspace $T_{\mu }^{c}D_{c}\subseteq T_{\mu }D_{c}$ defined by (32);*
*(c)*  *Let $U=\{u_{1},\cdots ,u_{c}\}$ denote an orthonormal basis of $C^{c}$ such that for every $\pi_{i}\in \Pi ,\;i\in \left[l\right]$, there are $u_{i_{1}},\cdots ,u_{i_{k}}\in U$ that form a basis of $\;\mathrm{range}(\pi_{i})$. Then, there is an inclusion of commutative subsets $D_{\Pi }↪D_{\Pi_{U}}$ that corresponds to an inclusion $S_{l}↪S_{c}$.*

**Proof.**  Appendix A.3.    □

Now, we establish that a restriction of the QSAF Equation (109) to the commutative product submanifold can be expressed in terms of the AF Equation (62). Analogous to the definition (96) of the product manifold $Q_{c}$, we set

(125) $$D_{\Pi ,c}=\underset{|V|\;\mathrm{factors}}{\underset{︸}{D_{\Pi }\times \cdots \times D_{\Pi }}}.$$

If Π is given by an orthonormal basis as in (124), we define the unitary matrices as

(126) $$\begin{array}{cc} U & =\left(u_{1},\cdots ,u_{c}\right)\in \mathrm{Un}\left(c\right), \end{array}$$

(127) $$\begin{array}{cc} U_{c} & =\underset{|V|\;\mathrm{block}-\mathrm{diagonal}\;\mathrm{entries}}{\underset{︸}{\mathrm{Diag}(U,\cdots ,U)}}. \end{array}$$

**Proposition** **10** (**invariance of** $D_{\Pi ,c}$). *Let* Π *and $D_{\Pi }$ be given according to Definition 2. Then, the following holds.*

*(i)*  *If $\mu \in D_{\Pi ,c}\subset Q_{c}$, then $R\left[\Omega [\mu ]\right]\in T_{\mu }D_{\Pi ,c}\subseteq T_{\mu }Q_{c}$.*
*(ii)*  *If $\Pi_{U}$ has the form (124), then*
  (128) $$R\left[\Omega [\mu ]\right]=U_{c}\mathrm{Diag}\left[R_{S}\left[\Omega S\right]\right]U_{c}^{*},$$
  where $S\in W_{c}$ is determined by $\mu_{i}=U\mathrm{Diag}\left(S_{i}\right)U^{*},\;i\in V$.

*In particular, the submanifold $D_{\Pi ,c}$ is preserved by the quantum state assignment flow.*

**Proof.**  Appendix A.3.    □

It remains to be verified that under suitable conditions on the data matrices $D_{i},\;i\in V$ that define the initial point of (109) by similarity mapping (Lemma 8), the quantum state assignment flow becomes the ordinary assignment flow.

**Corollary** **3** (**recovery of the AF by restriction**). *In the situation of Proposition 10, assume that all data matrices $D_{i},\;i\in V$ become diagonal in the same basis U, i.e.,*

(129) $$D_{i}=U\mathrm{Diag}\left(\lambda_{i}\right)U^{*},\;\lambda_{i}\in R^{c},\;i\in V.$$

*Then, the solution of the QSAF*

(130) $$\dot{\mu }=R_{\mu }\left[\Omega [\mu ]\right],\;\mu \left(0\right)=S\left(1_{Q_{c}}\right)$$

*is given by*

(131) $$\mu_{i}\left(t\right)=U\mathrm{Diag}\left(S_{i}\left(t\right)\right)U^{*},\;i\in V,$$

*where S(t) satisfies the ordinary AF equation*

(132) $$\dot{S}=R_{S}\left[\Omega S\right],\;S\left(0\right)=S\left(1_{W_{c}}\right),$$

*and the initial point is determined by the similarity map (56) evaluated at the barycenter $W=1_{W_{c}}$ with the vectors $\lambda_{i},\;i\in V$ as data points.*

**Proof.**  Appendix A.3.    □

## 5. Experiments and Discussion

In this section, we report academic experiments in order to illustrate the novelty of our approach. In comparison to the original formulation, our approach enables a continuous assignment without the need to specify explicitly prototypical labels beforehand. The experiments highlight the following properties of the novel approach, which extend the expressivity of the original assignment flow approach:

- Geometric *adaptive* feature vector averaging even when *uniform* weights are used (Section 5.2);
- Structure-preserving feature *patch* smoothing *without* accessing data at individual *pixels* (Section 5.3);
- Seamless incorporation of feature *encoding* using finite *frames* (Section 5.3).

In Section 6, we indicate the potential to represent spatial feature *context* via entanglement. However, working out the potential for various applications more thoroughly is beyond the scope of this paper.

### 5.1. Geometric Integration

In this section, we focus on the geometric integration of the reparameterized flow described by Equation (109). For a reasonable choice of a single-step-sized parameter, the scheme is accurate, stable and amenable to highly parallel implementations.

The e-geodesic from Proposition 4 constitutes a retraction [37] (Definition 4.1.1 and Proposition 5.4.1) onto the state manifold $Q_{c}$.

Consequently, the iterative step for updating $\mu_{t}\in Q_{c},\;t\in N_{0}$ and step size ϵ>0 is given by

(133) $$\begin{array}{cc} {\left(\mu_{t+1}\right)}_{i} & ={\left({\mathrm{Exp}}_{\mu_{t}}^{\left(e\right)}\left(\epsilon \;R_{\mu_{t}}\left[\Omega [\mu_{t}]\right]\right)\right)}_{i}=\left({\mathrm{Exp}}_{{\left(\mu_{t}\right)}_{i}}^{\left(e\right)}\circ R_{{\left(\mu_{t}\right)}_{i}}\left[\epsilon {\left(\Omega \left[\mu_{t}\right]\right)}_{i}\right]\right) \end{array}$$

(134) $$\begin{array}{cc} \; & \overset{\left(76\right)}{=}\exp_{{\left(\mu_{t}\right)}_{i}}\left(\epsilon {\left(\Omega \left[\mu_{t}\right]\right)}_{i}\right),\;\forall i\in V. \end{array}$$

for all i∈V. Using (77) and assuming

(135) $${\left(\mu_{t}\right)}_{i}=\Gamma \left({\left(A_{t}\right)}_{i}\right),\;\forall i\in V,$$

with $A_{t}\in T_{c}$, we obtain

(136) $$\begin{array}{cc} \Gamma \left({\left(A_{t+1}\right)}_{i}\right)\; & :=\exp_{{\left(\mu_{t}\right)}_{i}}\left(\epsilon {\left(\Omega \left[\mu_{t}\right]\right)}_{i}\right) \end{array}$$

(137) $$\begin{array}{cc} \; & =\Gamma \left(\Gamma^{-1}\left({\left(\mu_{t}\right)}_{i}\right)+\epsilon {\left(\Omega \left[\mu_{t}\right]\right)}_{i}\right) \end{array}$$

(138) $$\begin{array}{cc} \; & =\Gamma \left(\Gamma^{-1}\circ \Gamma \left({\left(A_{t}\right)}_{i}\right)+\epsilon {\left(\Omega \left[\mu_{t}\right]\right)}_{i}\right) \end{array}$$

(139) $$\begin{array}{cc} \; & =\Gamma \left({\left(A_{t}\right)}_{i}+\epsilon {\left(\Omega \left[\mu_{t}\right]\right)}_{i}\right),\;i\in V, \end{array}$$

and in view of (68) and (135), we conclude that

(140) $$A_{t+1}=A_{t}+\epsilon \Pi_{c,0}\Omega \left[\Gamma \left(A_{t}\right)\right].$$

**Remark** **5.** *We note that the numerical evaluation of the replicator operator (64) is not required. This makes the geometric integration scheme summarized by Algorithm 1 quite efficient.*

**Algorithm 1:**Geometric Integration Scheme

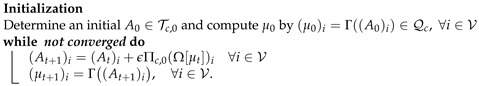



We list a few further implementation details below.

- A reasonable convergence criterion that measures how close the states are to a rank-one matrix is $|{\mathrm{tr}(\mu_{t})}_{i}-{\mathrm{tr}(\mu_{t}^{2})}_{i}|\leq \varepsilon ,\;\forall i\in V$;
- A reasonable range for the step size parameter is ϵ≤0.1;
- In order to remove spurious non-Hermitian numerical rounding errors, we replace each matrix $\left(\Omega {\left[\mu_{t}\right]}_{i}\right)$ with $\frac{1}{2}\left({\left(\Omega \left[\mu_{t}\right]\right)}_{i}+{\left(\Omega \left[\mu_{t}\right]\right)}_{i}^{*}\right)$;
- The constraint ρ=1 of (18) can be replaced by ρ=τ with any constant τ>1. This ensures that for larger matrix dimensions *c*, the entries of ρ vary in a reasonable numerical range and that the stability of the iterative updates.

Up to moderate matrix dimensions, such as c≤100, the matrix exponential in (66) can be computed using any of the basic established algorithms [38] (Chapter 10) or available solvers. In addition, depending on the size of the neighborhood $N_{i}$ induced by the weighted adjacency relation of the underlying graph in (95), Algorithm 1 can be implemented in a fine-grained parallel fashion.

### 5.2. Labeling 3D Data on Bloch Spheres

For the purpose of visual illustration, we consider the smoothing of 3D color vectors $d={\left(d_{1},d_{2},d_{3}\right)}^{⊤}$ interpreted as Bloch vectors, which parameterize density matrices [14] (Section 5.2).

(141) $$\rho =\rho \left(d\right)=\frac{1}{2}\left(I+d_{1}\left(\begin{array}{cc} 0 & 1\\ 1 & 0 \end{array}\right)+d_{2}\left(\begin{array}{cc} 0 & -i\\ i & 0 \end{array}\right)+d_{3}\left(\begin{array}{cc} 1 & 0\\ 0 & -1 \end{array}\right)\right)\in C^{2\times 2},\;∥d∥\leq 1.$$

**Figure 1:**
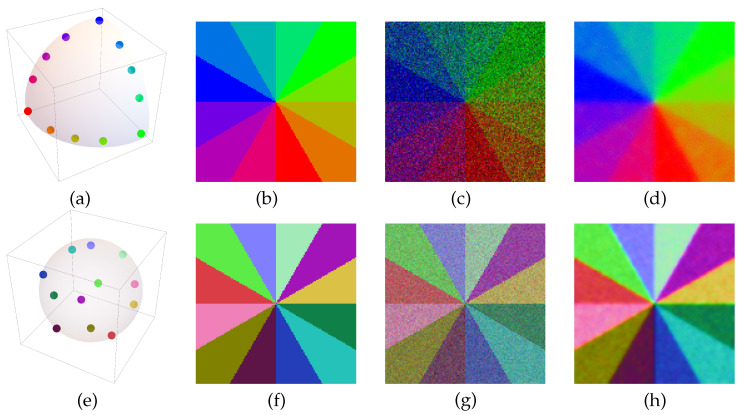
(**a**) A range of RGB unit color vectors in the positive orthant. (**b**) An image with data according to (**a**). (**c**) A noisy version of (**b**): each pixel i∈V displays a Bloch vector $d_{i}={\left(d_{i,1},d_{i,2},d_{i,3}\right)}^{⊤}$ defined by Equation (141) as an initial density matrix $\rho_{i}\left(0\right),\;i\in V$ of the QSAF. (**d**) The labels (pure states) generated by integrating the quantum state assignment flow using uniform weights. (**e**) The vectors depicted by (**a**) are replaced by the unit vectors corresponding to the vertices of the icosahedron centered at 0. (**f**–**h**) Analogous to (**b**–**d**), based on (**e**) instead of (**a**) and using the same noise level in (**g**). The colors in (**f**–**h**) visualize the Bloch vectors by RGB vectors that result from translating the sphere of (**e**) to the center $\frac{1}{2}{\left(1,1,1\right)}^{⊤}$ of the RGB cube and scaling it by $\frac{1}{2}$. We refer to the text for a discussion.

Pure states ρ correspond to unit vectors d,∥d∥=1, whereas vectors d,∥d∥<1 parameterize mixed states ρ. Given data $d_{i}={\left(d_{i,1},d_{i,2},d_{i,3}\right)}^{⊤},\;i\in V$ with $∥d_{i}∥\leq 1$, as illustrated by Figure 1 and explained in the caption, we initialized the QSAF at $\rho_{i}=\rho \left(d_{i}\right),\;i\in V$ and integrated the flow. Each integration step involves geometric state averaging across the graph, causing mixed states $\rho_{i}\left(t\right)=\rho \left(d_{i}\left(t\right)\right),\;i\in V$, which eventually converge towards pure states. Integration was stopped at time t=T, when $\min\{∥d_{i}\left(T\right)∥:i\in V\}\geq 0.999$. The resulting vectors $d_{i}\left(T\right)$ are visualized as explained in the caption of Figure 1. We point out that the two experiments discussed next are supposed to illustrate the behavior of the QSAF and the impact of the underlying geometry rather than constitute a contribution to the literature on the processing of color images.

Figure 1c shows a noisy version of the image in (b) used to initialize the quantum state assignment flow (QSAF). Panel (d) shows the labeled image, i.e., the assignment of a pure state (depicted as a Bloch vector) to each pixel of the input data (c). Although uniform weights were used and any prior information was absent, the result (d) demonstrates that the QSAF removes the noise and preserves the signal transition fairly well both for large-scale local image structure (away from the image center) and for small-scale local image structure (close to the image center). This behavior is quite unusual in comparison to traditional image denoising methods, which inevitably require *adaption* of regularization to the scale of local image structure. In addition, we note that noise removal is ‘perfect’ for the three extreme points (red, green and blue in panel (a)) but suboptimal only for the remaining non-extreme points.

Panels (f–h) show the same results when the data are encoded in a better way, as depicted by (e) using unit vectors not only on the positive orthant but on the whole unit sphere. These data are illustrated by RGB vectors that result from translating the unit sphere (e) to the center $\frac{1}{2}{\left(1,1,1\right)}^{⊤}$ of the RGB color cube ${\left[0,1\right]}^{3}$ and scaling it by $\frac{1}{2}$. This improved data encoding is clearly visible in panel (g), which displays the *same* noise level as shown in panel (c). Accordingly, noise removal while preserving signal structure at *all* local scales is more effectively achieved by the QSAF in (h) in comparison to (d).

### 5.3. Basic Image Patch Smoothing

**Figure 2:**
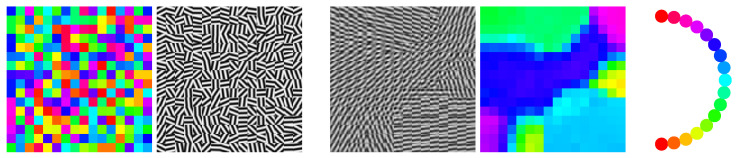
**Left pair:** A random collection of patches with oriented image structure. The colored image of each patch shows its orientation using the color code depicted by the rightmost panel. Each patch is represented by a rank-one matrix *D* in (89) obtained by vectorizing the patch and taking the tensor product. **Center pair:** The final state of the QSAF obtained by geometric integration with uniform weighting $\omega_{ik}=\frac{1}{|N_{i}|},\;\forall k\in N_{i},\;\forall i\in V$ of the nearest neighbor states. It represents an image partition but preserves image structure due to geometric smoothing of patches encoded by non-commutative state spaces.

Figure 2 shows an application of the QSAF to a *random* spatial arrangement (grid graph) of normalized patches, where each vertex represents a patch, not a pixel. Applying vectorization taking the tensor product with itself, each patch is represented as a pure state in terms of a rank-one matrix $D_{i}$ at the corresponding vertex i∈V, which constitutes the input data in the similarity mapping (100). Integrating the flow causes the non-commutative interaction of the associated state spaces $\rho_{i},\;i\in V$ through geometric averaging with uniform weights (95) until convergence towards pure states. The resulting patches are then simply given by the corresponding eigenvector, possibly after reversing the arbitrary sign of each eigenvector component, depending on the distance to the input patch.

The result shown in Figure 2 reveals an interesting behavior: structure-preserving patch smoothing without accessing explicitly individual pixels. In particular, the flow induces a *partition* of the patches without any prior assumption on the data.

**Figure 3:**
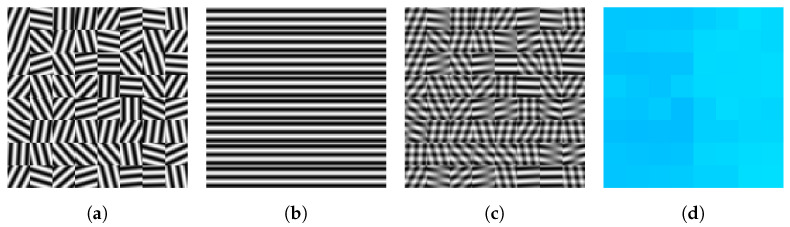
(**a**) A random collection of patches with oriented image structure. (**b**) A collection of patches with the same oriented image structure. (**c**) Pixel-wise mean of the patches (**a**,**b**) at each location. (**d**) The QSAF recovers a close approximation of (**b**) (color code: see Figure 2) by iteratively smoothing the states $\rho_{k},\;k\in N_{i}$ corresponding to (**c**) through geometric integration.

Figure 3 shows a variant of the scenario depicted in Figure 2 in order to demonstrate the ability to separate local image structure by geometric smoothing at the patch level in another way.

**Figure 4:**
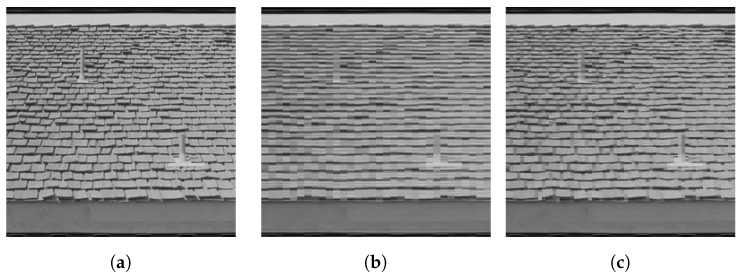
(**a**) A real image partitioned into patches of size 8×8 and 4×4 pixels, respectively. Each patch is represented as a pure state with respect to a Fourier frame (see text). Instead of the nearest neighbor adjacency on a regular grid, each patch is adjacent to its eight closest patches in the entire collection. Integrating the QSAF and decoding the resulting states (see text) yield (**b**) (8×8 patches) and (**c**) (4×4 patches), respectively. Result (**b**) illustrates the effect of smoothing at the patch level in the Fourier domain, whereas the smaller spatial scale used to compute (**c**) represents the input data fairly accurately, despite achieving significant data reduction.

Figure 4 generalizes the setup in two ways. First, patches were encoded using the harmonic frame given by the two-dimensional discrete Fourier matrix. Secondly, non-uniform weights $\omega_{ik}=e^{-\tau ∥P_{i}-P_{j}{∥}_{F}^{2}},\;\tau >0$ were used depending on the distance of adjacent patches $P_{i},P_{j}$.

Specifically, let $P_{i}$ denote the patch at vertex i∈V after removing the global mean and normalization using the Frobenius norm. Then, applying the FFT to each patch and vectorization formally with the discrete two-dimensional Fourier matrix $F_{2}=F⊗F$ (Kronecker product) followed by stacking the rows ${\hat{p}}_{i}=F_{2}\mathrm{vec}\left(P_{i}\right)$, the input data were defined as $D_{i}=F_{2}\mathrm{Diag}(-|{\hat{p}}_{i}{|}^{2})F_{2}^{*}$, where the squared magnitude ${\left|\cdot \right|}^{2}$ was computed component-wise. The flow yields were integrated again against pure states that were interpreted and decoded accordingly. The eigenvector was used as a multiplicative filter of the magnitude of the Fourier-transformed patch (keeping its phase), followed by rescaling of the norm and addition of the mean by approximating the original patch in terms of these two parameters.

The results shown in panels (**b**) and (**c**) of Figure 4 illustrate the effect of ‘geometric diffusion’ at the patch level through integration of the flow and how the input data are approximated depending on the chosen spatial scale (patch size), subject to significant data reduction.

## 6. Conclusions

We generalized the assignment flow approach for categorical distributions [2] to density matrices on weighted graphs. While the former flows assign each data point a label selected from a *finite* set, the latter assign each data point a generalized “label” from the *uncountable* submanifold of pure states.

**Figure 5:**
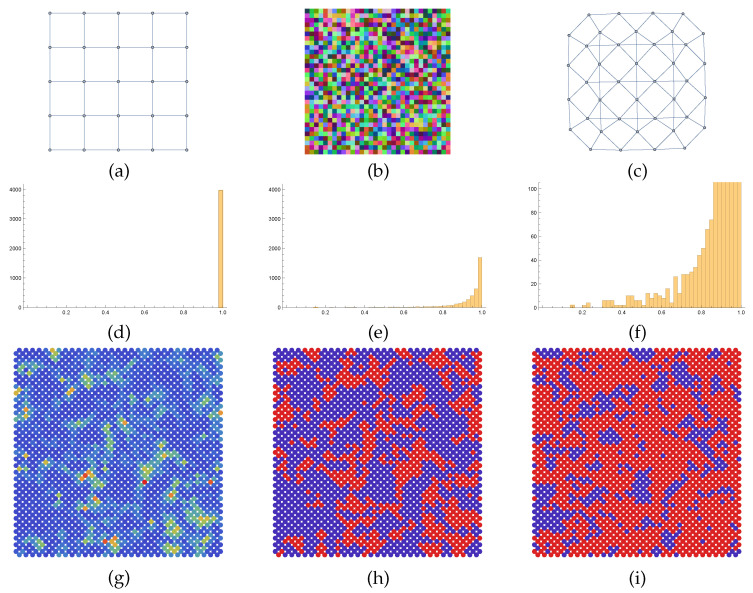
(**a**) A 5×5 grid graph. (**b**) Random Bloch vectors $d_{i}\in S^{2}\subset R^{3}$ (visualized using pseudocolor) defining states $\rho_{i}$ according to Equation (141) for each vertex of a 32×32 grid graph. (**c**) Line graph corresponding to (**a**). Each vertex corresponds to an edge ij of the graph (**a**) and an initially separable state $\rho_{ij}=\rho_{i}⊗\rho_{j}$. This defines a simple shallow tensor-network. The histograms display the norms of the Bloch vectors of the states ${}_{j}\left(\rho_{ij}\right)$ and ${}_{i}\left(\rho_{ij}\right)$ obtained by partially tracing out one factor for each state $\rho_{ij}$ indexed by a vertex ij of the line graph of the grid graph in (**b**). (**d**) Histogram showing that in the initial state, all states are separable, while (**e**,**f**) both display a histogram of the norms of all Bloch vectors after convergence of the quantum state assignment flow with uniform weights towards pure states. (**g**) Using the center coordinates of each edge of the grid graph (**b**), the entanglement represented by $\rho_{ij}$ is visualized by a disk and “heat map” colors (blue: low entanglement, red: large entanglement). For visual clarity, (**h**,**i**) again display the *same* information after thresholding, using two colors only: entangled states are marked with red when the norm of the Bloch vectors drops below the thresholds of 0.95 and 0.99, respectively, and otherwise with blue.

Various further directions of research are indicated by numerical experiments. This includes the unusual behavior of feature vector smoothing, which parameterizes complex-valued, non-commutative state spaces (Figure 1), the structure-preserving interaction of spatially indexed feature patches without accessing individual pixels (Figure 2 and Figure 3), the use of frames for signal representation and as observables whose expected values are governed by a quantum state assignment flow (Figure 4) and the representation of spatial correlations by entanglement and tensorization (Figure 5). Extending the representation of the original assignment flow in the broader framework of geometric mechanics to the novel quantum assignment flow approach as recently developed by [39] is another promising research project spurred by established concepts of mathematics and physics.

Based on these viewpoints, this paper adds a novel concrete approach based on information theory to the emerging literature on network design based on concepts from quantum mechanics, e.g., [40] and references therein. Our main motivation is the definition of a novel class of “neural ODEs” [41] in terms of the dynamical systems that generate a quantum state assignment flow. The layered architecture of a corresponding “neural network” is implicitly given by geometric integration. The inherent smoothness of the parameterization allows weight parameters to be learned from data. This will be explored in our future work, along the various lines of research indicated above.

## Figures and Tables

**Table 1:** Components of the the *Assignment Flow* approach and the corresponding components of the novel *Quantum State Assignment Flow approach*.

Assignment Flow (AF)	Quantum State AF (QSAF)
Single-vertex AF (Section 3.1)	Single-vertex QSAF (Section 4.2)
AF approach (Section 3.2)	QSAF approach (Section 4.3)
Riemannian gradient AF (Section 3.3)	Riemannian gradient QSAF (Section 4.4)
Recovery of the AF from the QSAF by restriction (Section 4.5)	

## Appendix A. Proofs

### Appendix A.1. Proofs of Section 2

**Proof** **Proposition** **1** We verify (13) and (14) by direct computation. For any $p\in S_{c}$,

(A1) $$\begin{array}{cc} R_{p}1_{c} & =\left(\mathrm{Diag}\left(p\right)-pp^{⊤}\right)1_{c}=p-\langle p,1_{c}\rangle p=0, \end{array}$$

(A2) $$\begin{array}{cc} R_{p}\pi_{c,0} & =R_{p}\left(I-1_{c}1_{S_{c}}^{⊤}\right)=R_{p}, \end{array}$$

(A3) $$\begin{array}{cc} \pi_{c,0}R_{p} & =\left(I-1_{c}1_{S_{c}}^{⊤}\right)R_{p}=R_{p}-\frac{1}{c}1_{c}{\left(R_{p}1_{c}\right)}^{⊤}=R_{p}. \end{array}$$

Next, we characterize the geometric role of $R_{p}$ and show (15). Let $p\in S_{c}$ be parameterized by the local coordinates

(A4) $$\begin{array}{cc} \bar{p} & =\varphi \left(p\right):={\left(p_{1},p_{2},\cdots ,p_{c-1}\right)}^{⊤}\in R_{++}^{c-1} \end{array}$$

(A5) $$\begin{array}{cc} p & =\varphi^{-1}\left(\bar{p}\right)={\left({\bar{p}}_{1},\cdots ,{\bar{p}}_{c-1},1-\langle 1_{c-1},\bar{p}\rangle \right)}^{⊤}\in S_{c}. \end{array}$$

Choosing the canonical basis $e_{1},\cdots ,e_{c}$ on $S_{c}\subset R^{c}$, we obtain a basis of the tangent space $T_{c,0}$

(A6) $$e_{j}-e_{c}=d\varphi^{-1}\left(e_{j}\right),\;j\in \left[c-1\right].$$

Using these vectors, a column of the matrix is

(A7) $$B:=\left(e_{1}-e_{c},\cdots ,e_{c-1}-e_{c}\right)=\left(\begin{array}{c} I_{c-1}\\ -1_{c-1}^{⊤} \end{array}\right)\in R^{c\times (c-1)},$$

one has for any $v\in T_{c,0}$

(A8) $$\begin{array}{cccc} v & =B\bar{v}=\left(\begin{array}{c} \bar{v}\\ v_{c} \end{array}\right)=\left(\begin{array}{c} \bar{v}\\ -\langle 1_{c-1},\bar{v}\rangle \end{array}\right), & \bar{v} & ={\left(v_{1},\cdots ,v_{c-1}\right)}^{⊤} \end{array}$$

(A9) $$\begin{array}{cccc} \bar{v} & =B^{†}v, & B^{†} & =\left(\begin{array}{cc} I_{c-1} & 0 \end{array}\right)\pi_{c,0}, \end{array}$$

where $B^{†}:={\left(B^{⊤}B\right)}^{-1}B^{⊤}$ denotes the Moore–Penrose generalized inverse of *B*. Substituting this parameterization and evaluating the metric (8) gives

(A10) $$\begin{array}{cc} g_{p}\left(u,v\right)\; & =\langle \bar{u},B^{⊤}\mathrm{Diag}{\left(p\right)}^{-1}B\bar{v}\rangle =\langle \bar{u},\left(\begin{array}{cc} I_{c-1} & -1_{c-1} \end{array}\right)\mathrm{Diag}{\left(p\right)}^{-1}\left(\begin{array}{c} I_{c-1}\\ -1_{c-1}^{⊤} \end{array}\right)\bar{v}\rangle \end{array}$$

(A11) $$\begin{array}{cc} \; & =\langle \bar{u},\left(\mathrm{Diag}{\left(\bar{p}\right)}^{-1}+\frac{1}{1-\langle 1_{c-1},\bar{p}\rangle }1_{c-1}1_{c-1}^{⊤}\right)\bar{v}\rangle \end{array}$$

(A12) $$\begin{array}{cc} \; & =:\langle \bar{u},G\left(\bar{p}\right)\bar{v}\rangle . \end{array}$$

Applying the Sherman–Morrison–Woodbury matrix inversion formula [42] (p. 9)

(A13) $${\left(A+xy^{⊤}\right)}^{-1}=A^{-1}-\frac{A^{-1}xy^{⊤}A^{-1}}{1+\langle y,A^{-1}x\rangle }$$

yields

(A14) $$\begin{array}{cc} G{\left(\bar{p}\right)}^{-1}\; & =\mathrm{Diag}\left(\bar{p}\right)-\frac{1}{1-\langle 1_{c-1},\bar{p}\rangle }\frac{\mathrm{Diag}\left(\bar{p}\right)1_{c-1}1_{c-1}^{⊤}\mathrm{Diag}\left(\bar{p}\right)}{1+\frac{1}{1-\langle 1_{c-1},\bar{p}\rangle }\langle 1_{c-1},\bar{p}\rangle } \end{array}$$

(A15) $$\begin{array}{cc} \; & =\mathrm{Diag}\left(\bar{p}\right)-\mathrm{Diag}\left(\bar{p}\right)1_{c-1}1_{c-1}^{⊤}\mathrm{Diag}\left(\bar{p}\right)=\mathrm{Diag}\left(\bar{p}\right)-\bar{p}\;{\bar{p}}^{⊤} \end{array}$$

(A16) $$\begin{array}{cc} \; & =R_{\bar{p}}. \end{array}$$

Let $v\in T_{c,0}$. Then, using the equations

(A17) $$\begin{array}{cc} p_{c} & \overset{\left(A5\right)}{=}1-\langle 1_{c-1},\bar{p}\rangle , \end{array}$$

(A18) $$\begin{array}{cc} R_{\bar{p}}1_{c-1} & =\bar{p}-\langle 1_{c-1},\bar{p}\rangle \bar{p}=p_{c}\bar{p}, \end{array}$$

we have

(A19) $$\begin{array}{cc} R_{p}v & =\left(\begin{array}{cc} R_{\bar{p}} & -p_{c}\bar{p}\\ -p_{c}{\bar{p}}^{⊤} & p_{c}-p_{c}^{2} \end{array}\right)\left(\begin{array}{c} \bar{v}\\ v_{c} \end{array}\right)=\left(\begin{array}{c} R_{\bar{p}}\bar{v}-v_{c}R_{\bar{p}}1_{c-1}\\ -\langle R_{\bar{p}}1_{c-1},\bar{v}\rangle +v_{c}p_{c}\langle 1_{c-1},\bar{p}\rangle \end{array}\right) \end{array}$$

(A20) $$\begin{array}{cc} \; & =\left(\begin{array}{c} R_{\bar{p}}\bar{v}\\ -\langle 1_{c-1},R_{\bar{p}}\bar{v}\rangle \end{array}\right)-v_{c}\left(\begin{array}{c} R_{\bar{p}}1_{c-1}\\ -\langle 1_{c-1},R_{\bar{p}}1_{c-1}\rangle \end{array}\right) \end{array}$$

(A21) $$\begin{array}{cc} \; & \overset{\left(A7\right)}{=}BR_{\bar{p}}\left(\bar{v}-v_{c}1_{c-1}\right). \end{array}$$

Now, consider any smooth function $f:S_{c}\to R$. Then,

(A22) $$\begin{array}{cc} \partial_{{\bar{p}}_{i}}\left(f\circ \varphi^{-1}\left(\bar{p}\right)\right)\; & =\sum_{j\in [c]}\partial_{j}f\left(p\right)\partial_{{\bar{p}}_{i}}\varphi^{-1}\left(\bar{p}\right)\overset{\left(A5\right)}{=}\partial_{i}f\left(p\right)-\partial_{c}f\left(p\right), \end{array}$$

(A23) $$\begin{array}{cc} \partial_{\bar{p}}\left(f\circ \varphi^{-1}\left(\bar{p}\right)\right)\; & =\bar{\partial f(p)}-\partial_{c}f\left(p\right)1_{c-1}. \end{array}$$

Comparing the last equation and (A21) shows that

(A24) $$R_{p}\partial f\left(p\right)=BR_{\bar{p}}\partial_{\bar{p}}\left(f\circ \varphi^{-1}\left(\bar{p}\right)\right)\overset{\left(A14\right)}{=}BG{\left(p\right)}^{-1}\partial_{\bar{p}}\left(f\circ \varphi^{-1}\left(\bar{p}\right)\right),$$

which proves (15). □

### Appendix A.2. Proofs of Section 3

**Proof** **of** **Lemma** **1.** Let $v\left(t\right)\in T_{c,0}$ be a smooth curve with $\dot{v}\left(t\right)=u$. Then,

(A25) $$\begin{array}{cc} \frac{d}{dt}\exp_{p}\left(v(t)\right)\; & =\frac{d}{dt}\frac{p\cdot e^{v(t)}}{\langle p,e^{v(t)}\rangle }=\frac{p\cdot u\cdot e^{v(t)}}{\langle p,e^{v(t)}\rangle }-\langle p,u\cdot e^{v(t)}\rangle \frac{p\cdot e^{v(t)}}{{\langle p,e^{v(t)}\rangle }^{2}} \end{array}$$

(A26) $$\begin{array}{cc} \; & =\exp_{p}\left(v(t)\right)\cdot u-\langle u,\exp_{p}\left(v(t)\right)\rangle \exp_{p}\left(v(t)\right)=R_{\exp_{p}\left(v\left(t\right)\right)}u. \end{array}$$

Similarly, for a smooth curve $p\left(t\right)\in S_{c}$ with $\dot{p}\left(t\right)=u$, one has

(A27) $$\begin{array}{cc} \; & \frac{d}{dt}\exp_{p(t)}\left(v\right)=\frac{d}{dt}\frac{p\left(t\right)\cdot e^{v}}{\langle p\left(t\right),e^{v}\rangle }=\frac{\dot{p}\left(t\right)\cdot e^{v}}{\langle p\left(t\right),e^{v}\rangle }-\langle \dot{p}\left(t\right),e^{v}\rangle \frac{p\left(t\right)\cdot e^{v}}{{\langle p\left(t\right),e^{v}\rangle }^{2}} \end{array}$$

(A28) $$\begin{array}{cc} \; & =\exp_{p(t)}\left(v\right)\cdot \frac{u}{p(t)}-\langle \frac{u}{p(t)},\exp_{p(t)}\left(v\right)\rangle \exp_{p(t)}\left(v\right)=R_{\exp_{p(t)}\left(v\right)}\frac{u}{p(t)}. \end{array}$$

□

**Proof** **of** **Theorem** **1.** Let

(A29) $$q\left(t\right)=L_{p(t)}\left(D\right)$$

where p(t) solves (45). Using (43), (47) and (A29), we obtain

(A30) $$\dot{q}=d_{p}L_{p(t)}\left(D\right)\left[\dot{p}\left(t\right)\right]=R_{q(t)}\left(\frac{\dot{p}\left(t\right)}{p(t)}\right)\overset{\left(45\right)}{=}R_{q(t)}\left(q\left(t\right)-\langle p\left(t\right),q\left(t\right)\rangle 1_{c}\right)\overset{\left(13\right)}{=}R_{q(t)}q\left(t\right),$$

which shows (49). Then, $p\left(t\right)=\exp_{1_{S_{c}}}\left(r\left(t\right)\right)$, and differentiating (50) yields $r\left(t\right)=\int_{0}^{t}q\left(\tau \right)d\tau$

(A31) $$\dot{p}\left(t\right)\overset{\left(46\right)}{=}R_{\exp_{1_{S_{c}}}\left(r\left(t\right)\right)}\dot{r}\left(t\right)\overset{\left(50\right)}{=}R_{p(t)}q\left(t\right)\overset{\left(A29\right)}{=}R_{p(t)}L_{p(t)}\left(D\right),$$

which proves the equivalence of (45), (48) and (49). □

**Proof** **of** **Corollary** **1.** The solution p(t) to (45) is given by (48) and (49). Proposition 1 and Equation (15) show that (49) is the Riemannian ascent flow of the function $S_{c}∋q\mapsto \frac{1}{2}{∥q∥}^{2}$. The stationary points satisfy

(A32) $$R_{q}q={(q-∥q∥}^{2})\cdot q=0$$

and form the set

(A33) $$Q^{*}:=\left\{q^{*}=\frac{1}{|J^{*}|}\sum_{j\in J^{*}}e_{j}:J^{*}\subseteq \left[c\right]\right\}.$$

The case of $J^{*}=\left[c\right]$, i.e., $q^{*}=1_{S_{c}}$, can be ruled out if $\frac{D}{\langle 1_{c},D\rangle }\neq S_{c}$, which is always the case in practice, where *D* corresponds to real data (measurement and observation). The global maxima correspond to the vertices of $\Delta_{c}={\bar{S}}_{c}$, i.e., $|J^{*}|=1$. The remaining stationary points are local maxima and degenerate, since vectors *D* with non-unique minimal components form a negligible null set. In any case, $\lim_{t\to \infty }p\left(t\right)\overset{\left(50\right)}{=}\lim_{t\to \infty }q\left(t\right)=q^{*}$, depending on the index set $J^{*}$ determined by *D*. □

### Appendix A.3. Proofs of Section 4

**Proof** **of** **Proposition** **2.** The Riemannian gradient is defined by [43] (p. 337)

(A34) $$\begin{array}{cc} 0 & =df\left[X\right]-g_{\rho }\left({\mathrm{grad}}_{\rho }f,X\right)\overset{\left(29\right)}{=}\langle \partial f,X\rangle -\langle T_{\rho }\left[{\mathrm{grad}}_{\rho }f\right],X\rangle \end{array}$$

(A35) $$\begin{array}{cc} \; & =\langle \partial f-T_{\rho }\left[{\mathrm{grad}}_{\rho }f\right],X\rangle ,\;\forall X\in H_{c,0}. \end{array}$$

Choosing the parameterization $X=Y-\mathrm{tr}(Y)I\in H_{c,0}$ with $Y\in H_{c}$, we further obtain

(A36) $$\begin{array}{cc} 0 & =\langle \partial f-T_{\rho }\left[{\mathrm{grad}}_{\rho }f\right],Y\rangle -\mathrm{tr}(Y)\mathrm{tr}(\partial f-T_{\rho }\left[{\mathrm{grad}}_{\rho }f\right]) \end{array}$$

(A37) $$\begin{array}{cc} \; & =\langle \partial f-T_{\rho }\left[{\mathrm{grad}}_{\rho }f\right]-\mathrm{tr}(\partial f-T_{\rho }\left[{\mathrm{grad}}_{\rho }f\right])I,Y\rangle ,\;\forall Y\in H_{c}. \end{array}$$

The left factor must vanish. Applying linear mapping $T_{\rho }^{-1}$ and solving for ${\mathrm{grad}}_{\rho }f$ yields

(A38) $${\mathrm{grad}}_{\rho }f=T_{\rho }^{-1}\left[\partial f\right]-\left(\partial f-T_{\rho }\left[{\mathrm{grad}}_{\rho }f\right]\right)T_{\rho }^{-1}\left[I\right].$$

Since ${\mathrm{grad}}_{\rho }f\in H_{c,0}$, taking the trace on both sides and using $T_{\rho }^{-1}\left[I\right]=\mathrm{tr}\rho =1$ yields

(A39) $$0=\mathrm{tr}T_{\rho }^{-1}\left[\partial f\right]-\mathrm{tr}\partial f+\mathrm{tr}T_{\rho }\left[{\mathrm{grad}}_{\rho }f\right].$$

Substituting the last two summands into the previous equation yields

(A40) $$\begin{array}{cc} {\mathrm{grad}}_{\rho }f\; & =T_{\rho }^{-1}\left[\partial f\right]-\left(\mathrm{tr}T_{\rho }^{-1}\left[\partial f\right]\right)\rho \end{array}$$

(A41) $$\begin{array}{cc} \; & =T_{\rho }^{-1}\left[\partial f\right]-\langle \rho ,\partial f\rangle \rho , \end{array}$$

where the last equation follows from (30). □

**Proof** **of** **Lemma** **2.** The equation $\Pi_{c,0}\circ R_{\rho }=R_{\rho }$ follows from $R_{\rho }\left[X\right]\in H_{c,0}$; hence,

(A42) $$\mathrm{tr}R_{\rho }\left[X\right]\overset{\left(64\right)}{=}\mathrm{tr}T_{\rho }^{-1}\left[X\right]-\langle \rho ,X\rangle \mathrm{tr}\rho \overset{\left(30\right)}{=}\langle \rho ,X\rangle -\langle \rho ,X\rangle =0.$$

Thus,

(A43) $$\begin{array}{cc} \Pi_{c,0}\circ R_{\rho }\left[X\right]\; & =R_{\rho }\left[X\right]=R_{\rho }\left[X\right]-\frac{\mathrm{tr}X}{c}\left(\rho -\underset{=1}{\underset{︸}{\langle \rho ,I\rangle }}\rho \right) \end{array}$$

(A44) $$\begin{array}{cc} \; & =R_{\rho }\left[X\right]-\frac{\mathrm{tr}X}{c}R_{\rho }\left[I\right]=R_{\rho }\left[X-\frac{\mathrm{tr}X}{c}I\right]\overset{\left(24\right)}{=}R_{\rho }\circ \Pi_{c,0}\left[X\right]. \end{array}$$

□

**Proof** **of** **Lemma** **3.** Using (24), we compute

(A45) $$\exp_{m}\left(\Pi_{c,0}\left[Z\right]\right)=\exp_{m}\left(Z-\frac{\mathrm{tr}Z}{c}I\right)=e^{\frac{Z}{c}}\exp_{m}\left(Z\right),$$

where the last equation holds, since *Z* and *I* commute. Substitution into (66) cancels the scalar factor $e^{\frac{\mathrm{tr}Z}{c}}$ and shows (68). □

**Proof** **of** **Proposition** **3.** We show $\Gamma \circ \Gamma^{-1}={\mathrm{id}}_{D_{c}}$ and $\Gamma^{-1}\circ \Gamma ={\mathrm{id}}_{H_{c,0}}$. As for the first relation, we compute

(A46) $$\begin{array}{cc} \Gamma \circ \Gamma^{-1}\left(\rho \right)\; & =\exp_{m}\left(\Gamma^{-1}\left(\rho \right)-\psi \left(\Gamma^{-1}\left(\rho \right)\right)I\right) \end{array}$$

(A47) $$\begin{array}{cc} \; & =\exp_{m}\left(\log_{m}\rho -\frac{\mathrm{tr}(\log_{m}\rho )}{c}I-\log(\mathrm{tr}\exp_{m}\left(\log_{m}\rho -\frac{\mathrm{tr}(\log_{m}\rho )}{c}I\right))I\right) \end{array}$$

and since $\log_{m}\rho$ and *I* commute,

(A48) $$\begin{array}{cc} \; & =\exp_{m}\left(\log_{m}\rho -\frac{\mathrm{tr}(\log_{m}\rho )}{c}I-\log\mathrm{tr}\left(e^{-\frac{1}{c}\mathrm{tr}(\log_{m}\rho )}\rho \right)I\right) \end{array}$$

(A49) $$\begin{array}{cc} \; & \overset{\mathrm{tr}\rho =1}{=}\exp_{m}\left(\log_{m}\rho \right) \end{array}$$

(A50) $$\begin{array}{cc} \; & =\rho . \end{array}$$

As for the second relation, we compute

(A51) $$\begin{array}{cc} \Gamma^{-1}\circ \Gamma \left(X\right)\; & =\Pi_{c,0}\left[\log_{m}\circ \Gamma \left(X\right)\right]=\Pi_{c,0}\left[\log_{m}\circ \exp_{m}\left(X-\psi (X)I\right)\right] \end{array}$$

(A52) $$\begin{array}{cc} \; & =\Pi_{c,0}\left[X\right]-\psi \left(X\right)\Pi_{c,0}\left[I\right]=\Pi_{c,0}\left[X\right] \end{array}$$

(A53) $$\begin{array}{cc} \; & =X, \end{array}$$

since $X\in H_{c,0}$ by assumption. □

**Proof** **of** **Lemma** **4.** In view of Definition (66) of Γ, we compute using the chain rule

(A54) $$\begin{array}{cc} d\Gamma (H)[Y]\; & =\frac{d}{dt}\exp_{m}\left(H+tY-\psi (H+tY)I\right){|}_{t=0} \end{array}$$

(A55) $$\begin{array}{cc} \; & =d\exp_{m}\left(H-\psi (H)I\right)\left[Y-d\psi (H)[Y]I\right] \end{array}$$

(A56) $$\begin{array}{cc} \; & \overset{\left(28\right)}{=}T_{\rho }^{-1}\left[Y-d\psi (H)[Y]I\right]. \end{array}$$

Furthermore,

(A57) $$\begin{array}{cc} d\psi (H)[Y]\; & \overset{\left(67\right)}{=}\frac{1}{\mathrm{tr}\exp_{m}\left(H\right)}\mathrm{tr}(d\exp_{m}\left(H\right)\left[Y\right]) \end{array}$$

(A58) $$\begin{array}{cc} \; & \overset{\left(28\right)}{=}\frac{1}{\mathrm{tr}\exp_{m}\left(H\right)}\mathrm{tr}\left(T_{\exp_{m}\left(H\right)}^{-1}\left[Y\right]\right),\;\exp_{m}\left(H\right)\overset{\left(66\right)}{=}(\mathrm{tr}\exp_{m}\left(H\right))\Gamma \left(H\right) \end{array}$$

(A59) $$\begin{array}{cc} \; & \overset{\left(30\right)}{=}\frac{1}{\mathrm{tr}\exp_{m}\left(H\right)}\langle \exp_{m}\left(H\right),Y\rangle \end{array}$$

(A60) $$\begin{array}{cc} \; & \overset{\left(66\right)}{=}\langle \Gamma \left(H\right),Y\rangle =\langle \rho ,Y\rangle , \end{array}$$

where the last equation follows from the assumption that ρ=Γ(H). Substitution into (A54) yields (70). Regarding (71), using expression (69) for $\Gamma^{-1}$, we compute

(A61) $$\begin{array}{cc} d\Gamma^{-1}\left(\rho \right)\left[X\right]\; & =\Pi_{c,0}\circ d\log_{m}\left(\rho \right)\left[X\right] \end{array}$$

(A62) $$\begin{array}{cc} \; & \overset{\left(27\right)}{=}\Pi_{c,0}\circ T_{\rho }\left[X\right], \end{array}$$

which verifies (71). □

**Proof** **of** **Proposition** **4.** The e-geodesic connecting the two points $Q,R\in D_{c}$ is given by [24] (Section V)

(A63) $$\Gamma \left(K+tA\right),\;t\in \left[0,1\right],\;K=\log_{m}Q,\;A=\log_{m}R-\log_{m}Q.$$

Setting $\Gamma^{-1}\left(\rho \right)=\Pi_{c,0}\left[K\right]$ and $T_{\rho }\left[X\right]=A$ yields (74), since the orthogonal projections $\Pi_{c,0}$ onto $H_{c,0}$ are also implicitly carried out in (A63) due to Lemma 3. Expression (73) is equal to (74) due to (71). It remains to be verified that the geodesic emanates at ρ in the direction of *X*. We compute

(A64) $$\begin{array}{cc} \gamma_{\rho ,X}^{\left(e\right)}\left(0\right)\; & =\Gamma (\Gamma^{-1}\left(\rho \right))=\rho \end{array}$$

(A65) $$\begin{array}{cc} \frac{d}{dt}\gamma_{\rho ,X}^{\left(e\right)}\left(0\right)\; & =\frac{d}{dt}\Gamma \left(\Gamma^{-1}\left(\rho \right)+td\Gamma^{-1}\left(\rho \right)\left[X\right]\right){|}_{t=0} \end{array}$$

(A66) $$\begin{array}{cc} \; & =d\Gamma \left(\Gamma^{-1}\left(\rho \right)\right)\left[d\Gamma^{-1}\left(\rho \right)\left[X\right]\right]=\mathrm{id}\left[X\right]=X. \end{array}$$

□

**Proof** **of** **Corollary** **2.** Setting

(A67) $$\mu ={\mathrm{Exp}}_{\rho }^{\left(e\right)}\left(X\right)\overset{\left(73\right)}{=}\Gamma \left(\Gamma^{-1}\left(\rho \right)+d\Gamma^{-1}\left(\rho \right)\left[X\right]\right)$$

we solve for *X*,

(A68) $$\begin{array}{cc} \Gamma^{-1}\left(\mu \right)\; & =\Gamma^{-1}\left(\rho \right)+d\Gamma^{-1}\left(\rho \right)\left[X\right] \end{array}$$

(A69) $$\begin{array}{cc} d\Gamma^{-1}\left(\rho \right)\left[X\right]\; & =\Gamma^{-1}\left(\mu \right)-\Gamma^{-1}\left(\rho \right) \end{array}$$

(A70) $$\begin{array}{cc} X & =d\Gamma \left(\Gamma^{-1}\left(\rho \right)\right)[\Gamma^{-1}\left(\mu \right)-\Gamma^{-1}\left(\rho \right)], \end{array}$$

which shows (75) and where $d\Gamma {\left(\Gamma^{-1}\left(\rho \right)\right)}^{-1}=d\Gamma^{-1}\left(\rho \right)$ was used to obtain the last equation. □

**Proof** **of** **Lemma** **5.** We compute

(A71) $$\begin{array}{cc} {\mathrm{Exp}}_{\rho }^{\left(e\right)}\circ R_{\rho }\left[X\right]\; & \overset{\left(73\right)}{=}\Gamma \left(\Gamma^{-1}\left(\rho \right)+\Pi_{c,0}\circ T_{\rho }\left[R_{\rho }\left[X\right]\right]\right) \end{array}$$

(A72) $$\begin{array}{cc} \; & \overset{\left(64\right)}{=}\Gamma \left(\Gamma^{-1}\left(\rho \right)+\Pi_{c,0}\circ T_{\rho }\left[T_{\rho }^{-1}\left[X\right]-\langle \rho ,X\rangle \rho \right]\right) \end{array}$$

(A73) $$\begin{array}{cc} \; & \overset{\rho =T^{-1}\left[I\right]}{=}\Gamma \left(\Gamma^{-1}\left(\rho \right)+\Pi_{c,0}\left[X-\langle \rho ,X\rangle I\right]\right) \end{array}$$

(A74) $$\begin{array}{cc} \; & =\Gamma \left(\Gamma^{-1}\left(\rho \right)+X\right) \end{array}$$

and omit the projection map $\Pi_{c,0}$ in the last equation due to Lemma 2 or Lemma 3. □

**Proof** **of** **Lemma** **6.** We compute

(A75) $$\begin{array}{cc} d\exp_{\rho }\left(X\right)\left[Y\right]\; & \overset{\left(77\right)}{=}d\Gamma (\Gamma^{-1}\left(\rho \right)+X)\left[Y\right]\overset{\left(70\right)}{=}T_{\exp_{\rho }\left(X\right)}^{-1}\left[Y-\langle \exp_{\rho }\left(X\right),Y\rangle I\right] \end{array}$$

(A76) $$\begin{array}{cc} \; & \overset{T_{\rho }^{-1}\left[I\right]=\rho }{=}T_{\exp_{\rho }\left(X\right)}^{-1}\left[Y\right]-\langle \exp_{\rho }\left(X\right),Y\rangle \exp_{\rho }\left(X\right) \end{array}$$

(A77) $$\begin{array}{cc} \; & \overset{\left(64\right)}{=}R_{\exp_{\rho }\left(X\right)}\left[Y\right]. \end{array}$$

□

**Proof** **of** **Lemma** **7.** We compute

(A78) $$\begin{array}{cc} L_{\rho }\left(D\right) & =\exp_{\rho }\left(-\Pi_{c,0}\left[D\right]\right)\overset{\begin{array}{c} \left(65\right)\\ \left(76\right) \end{array}}{=}\exp_{\rho }\left(-D\right) \end{array}$$

(A79) $$\begin{array}{cc} \; & \overset{\left(77\right)}{=}\Gamma \left(\Gamma^{-1}\left(\rho \right)-D\right)\overset{\left(69\right)}{=}\Gamma \left(\Pi_{c,0}\left[\log_{m}\rho \right]-D\right) \end{array}$$

(A80) $$\begin{array}{cc} \; & \overset{\left(92\right)}{=}\Gamma \left(Q\left(\log_{m}\Lambda_{\rho }\right)Q^{⊤}-\frac{1}{c}\left(\mathrm{tr}\Lambda_{\rho }\right)I_{c}-Q\Lambda_{D}Q^{⊤}\right) \end{array}$$

(A81) $$\begin{array}{cc} \; & \overset{\left(66\right)}{=}Q\frac{\mathrm{Diag}(e^{\log\lambda_{\rho }-\langle 1_{c},\lambda_{\rho }\rangle 1_{S_{c}}-\lambda_{D}})}{\langle 1_{c},e^{\log\lambda_{\rho }-\langle 1_{c},\lambda_{\rho }\rangle 1_{S_{c}}-\lambda_{D}}\rangle }Q^{⊤}=Q\mathrm{Diag}\left(\frac{e^{\log\lambda_{\rho }-\lambda_{D}}}{\langle 1_{c},e^{\log\lambda_{\rho }-\lambda_{D}}\rangle }\right)Q^{⊤} \end{array}$$

(A82) $$\begin{array}{cc} \; & \overset{\left(44\right)}{=}Q\mathrm{Diag}\left(\exp_{\lambda_{\rho }}\left(-\lambda_{D}\right)\right)Q^{⊤}. \end{array}$$

□

**Proof** **of** **Proposition** **5.** Writing $\rho_{0}=\rho \left(0\right)=1_{D_{c}}$ and $\mathrm{Diag}\left(\lambda_{D}\right)=\Lambda_{D}$, we have

$$L_{\rho_{0}}\left(D\right)=Q\mathrm{Diag}\left(\exp_{1_{S_{c}}}\left(-\lambda_{D}\right)\right)Q^{⊤}$$

according to Lemma 7 and

(A83) $$\begin{array}{cc} \dot{\rho }\left(0\right)\; & \overset{\left(91\right)}{=}T_{\rho_{0}}^{-1}\left[L_{\rho_{0}}\left(D\right)\right]-\langle \rho_{0},L_{\rho_{0}}\left(D\right)\rangle \rho_{0} \end{array}$$

(A84) $$\begin{array}{cc} \; & \overset{\left(28\right)}{=}\int_{0}^{1}{\left(\frac{1}{c}I_{c}\right)}^{1-s}Q\mathrm{Diag}\left(\exp_{1_{S_{c}}}\left(-\lambda_{D}\right)\right)Q^{⊤}{\left(\frac{1}{c}I_{c}\right)}^{s}ds \end{array}$$

(A85) $$\begin{array}{cc} & \;\;-\frac{1}{c^{2}}\underset{=1}{\mathrm{tr}\underset{︸}{\left(Q\mathrm{Diag}\left(\exp_{1_{S_{c}}}\left(-\lambda_{D}\right)\right)Q^{⊤}\right)}}I_{c} \end{array}$$

(A86) $$\begin{array}{cc} \; & =\frac{1}{c}Q\mathrm{Diag}\left(\exp_{1_{S_{c}}}\left(-\lambda_{D}\right)\right)Q^{⊤}-\frac{1}{c^{2}}I_{c} \end{array}$$

(A87) $$\begin{array}{cc} \; & =\frac{1}{c}Q\mathrm{Diag}\left(\exp_{1_{S_{c}}}\left(-\lambda_{D}\right)-1_{S_{c}}\right)Q^{⊤}. \end{array}$$

Comparing this equation to the single-vertex flow (45) at time t=0,

(A88) $$\begin{array}{cc} \dot{p}\left(0\right) & =\frac{1}{c}\left(\exp_{1_{S_{c}}}\left(-D\right)-\frac{1}{c}\underset{=1}{\underset{︸}{\langle 1_{c},\exp_{1_{S_{c}}}\left(-D\right)\rangle }}1_{c}\right)=\frac{1}{c}\left(\exp_{1_{S_{c}}}\left(-D\right)-1_{S_{c}}\right) \end{array}$$

shows that

(A89) $$\dot{\rho }\left(0\right)=Q\mathrm{Diag}\left({\dot{\lambda }}_{\rho }\left(0\right)\right)Q^{⊤},$$

that is

(A90) $$\begin{array}{cc} \rho (t) & =Q\lambda_{\rho }\left(t\right)Q^{⊤}, \end{array}$$

where λ(t) solves the single-vertex assignment flow Equation (45) of the form

(A91) $$\begin{array}{cc} {\dot{\lambda }}_{\rho } & =R_{\lambda_{\rho }}L_{\lambda_{\rho }}\left(\lambda_{D}\right). \end{array}$$

Corollary 1 completes the proof. □

**Proof** **of** **Lemma** **8.** Let

(A92) $$H_{i}=\Gamma^{-1}\left(\rho_{i}\right)\overset{\left(69\right)}{=}\Pi_{c,0}\log_{m}\rho_{i},\;i\in V.$$

Then,

(A93) $$\begin{array}{cc} {\left({\mathrm{Exp}}_{\rho_{i}}^{\left(e\right)}\right)}^{-1}\left(L_{\rho_{k}}\left(D_{k}\right)\right)\; & \overset{\begin{array}{c} \left(89\right)\\ \left(77\right) \end{array}}{=}{\left({\mathrm{Exp}}_{\rho_{i}}^{\left(e\right)}\right)}^{-1}\circ \Gamma \left(\Gamma^{-1}\left(\rho_{k}\right)-D_{k}\right) \end{array}$$

(A94) $$\begin{array}{cc} \; & \overset{\left(75\right)}{=}d\Gamma \left(\Gamma^{-1}\left(\rho_{i}\right)\right)\left[\Gamma^{-1}\left(\rho_{k}\right)-D_{k}-\Gamma^{-1}\left(\rho_{i}\right)\right] \end{array}$$

(A95) $$\begin{array}{cc} \; & =d\Gamma \left(H_{i}\right)\left[H_{k}-D_{k}-H_{i}\right]. \end{array}$$

Substituting this expression into (100) yields

(A96) $$\begin{array}{cc} S_{i}\left(\rho \right)\; & \overset{\begin{array}{c} \left(73\right)\\ \left(95\right) \end{array}}{=}\Gamma \left(H_{i}+\underset{=I}{\underset{︸}{d\Gamma^{-1}\left(\rho_{i}\right)\circ d\Gamma \left(H_{i}\right)}}\left[\sum_{k\in N_{i}}\omega_{ik}\left(H_{k}-D_{k}\right)-H_{i}\right]\right) \end{array}$$

(A97) $$\begin{array}{cc} \; & =\Gamma \left(\sum_{k\in N_{i}}\omega_{ik}\left(H_{k}-D_{k}\right)\right). \end{array}$$

Substituting (A92) and omitting the projection map $\Pi_{c,0}$ due to Lemma 3 yields (101). □

**Proof** **of** **Proposition** **6.** Substituting as in the proof of Lemma 8, we obtain

(A98) $$\begin{array}{cc} 0 & =d\Gamma \left(\Gamma^{-1}\left(\bar{\rho }\right)\right)\left[\sum_{k\in N_{i}}\omega_{ik}\left(\Pi_{c,0}\log_{m}\rho_{k}-D_{k}\right)-\Gamma^{-1}\left(\bar{\rho }\right)\right]. \end{array}$$

Since dΓ is one-to-one, the expression inside the brackets must vanish. Solving for $\bar{\rho }$ and omitting the projection map $\Pi_{c,0}$ due to Lemma 3 yields (101). □

**Proof** **of** **Proposition** **7.** Let ρ(t) solve (104) and denote the argument of the replicator operator $R_{\rho }$ on the right-hand side by

(A99) $$\mu \left(t\right):=S\left(\rho (t)\right),$$

which yields (108) and (104), respectively, whereas (109) remains to be verified. Differentiation yields

(A100) $$\begin{array}{cc} {\dot{\mu }}_{i} & =dS_{i}\left(\rho \right)\left[\dot{\rho }\right] \end{array}$$

(A101) $$\begin{array}{cc} \; & \overset{\begin{array}{c} \left(101\right)\\ \left(27\right) \end{array}}{=}d\Gamma \left(\sum_{k\in N_{i}}\omega_{ik}\left(\log_{m}\rho_{k}-D_{k}\right)\right)\left[\sum_{k\in N_{i}}\omega_{ik}T_{\rho_{k}}\left[{\dot{\rho }}_{k}\right]\right] \end{array}$$

(A102) $$\begin{array}{cc} \; & \overset{\left(101\right)}{=}d\Gamma \left(\Gamma^{-1}(S_{i}\left(\rho \right)\right))\left[\sum_{k\in N_{i}}\omega_{ik}T_{\rho_{k}}\left[{\dot{\rho }}_{k}\right]\right] \end{array}$$

(A103) $$\begin{array}{cc} \; & \overset{\left(70\right)}{=}T_{S_{i}\left(\rho \right)}^{-1}\left[\sum_{k\in N_{i}}\omega_{ik}T_{\rho_{k}}\left[{\dot{\rho }}_{k}\right]-\langle S_{i}\left(\rho \right),\sum_{k\in N_{i}}\omega_{ik}T_{\rho_{k}}\left[{\dot{\rho }}_{k}\right]\rangle I\right] \end{array}$$

(A104) $$\begin{array}{cc} \; & \overset{T_{\rho }^{-1}\left[I\right]=\rho }{=}T_{S_{i}\left(\rho \right)}^{-1}\left[\sum_{k\in N_{i}}\omega_{ik}T_{\rho_{k}}\left[{\dot{\rho }}_{k}\right]\right]-\langle S_{i}\left(\rho \right),\sum_{k\in N_{i}}\omega_{ik}T_{\rho_{k}}\left[{\dot{\rho }}_{k}\right]\rangle S_{i}\left(\rho \right) \end{array}$$

(A105) $$\begin{array}{cc} \; & \overset{\left(64\right)}{=}R_{S_{i}\left(\rho \right)}\left[\sum_{k\in N_{i}}\omega_{ik}T_{\rho_{k}}\left[{\dot{\rho }}_{k}\right]\right]\overset{\left(A64\right)}{=}\sum_{k\in N_{i}}\omega_{ik}R_{\mu_{i}}\left[T_{\rho_{k}}\left[{\dot{\rho }}_{k}\right]\right] \end{array}$$

(A106) $$\begin{array}{cc} \; & \overset{\left(108\right)}{=}\sum_{k\in N_{i}}\omega_{ik}R_{\mu_{i}}\left[T_{\rho_{k}}\left[R_{\rho_{k}}\left[\mu_{k}\right]\right]\right] \end{array}$$

(A107) $$\begin{array}{cc} \; & \overset{\left(64\right)}{=}\sum_{k\in N_{i}}\omega_{ik}R_{\mu_{i}}\left[T_{\rho_{k}}\left[T_{\rho_{k}}^{-1}\left[\mu_{k}\right]-\langle \rho_{k},\mu_{k}\rangle T_{\rho_{k}}^{-1}\left[I\right]\right]\right] \end{array}$$

(A108) $$\begin{array}{cc} \; & =\sum_{k\in N_{i}}\omega_{ik}R_{\mu_{i}}\left[\mu_{k}-\langle \rho_{k},\mu_{k}\rangle I\right]\overset{\left(65\right)}{=}\sum_{k\in N_{i}}\omega_{ik}R_{\mu_{i}}\left[\mu_{k}\right] \end{array}$$

(A109) $$\begin{array}{cc} \; & \overset{\left(107\right)}{=}R_{\mu_{i}}\left[\Omega {\left[\mu \right]}_{i}\right]. \end{array}$$

The initial condition for ρ is given by (104). The initial condition for μ follows from (A99). □

**Proof** **of** **Proposition** **8.** We compute using (110) and (111)

(A110) $$\begin{array}{cc} J(\mu )\; & =-\frac{1}{2}\sum_{j\in V}\langle \mu_{j},\sum_{k\in N_{j}}\omega_{jk}\mu_{k}\rangle \end{array}$$

(A111) $$\begin{array}{cc} \partial_{\mu_{i}}J\left(\mu \right)\; & =-\frac{1}{2}\left(\sum_{k\in N_{i}}\omega_{ik}\mu_{k}+\sum_{j\in N_{i}}\omega_{ji}\mu_{j}\right)\overset{\left(110\right)}{=}-\Omega {\left[\mu \right]}_{i}. \end{array}$$

Consequently, according to Proposition 2 and (64),

(A112) $$-{\mathrm{grad}}_{\mu_{i}}J\left(\mu \right)=-R_{\mu_{i}}\left[\partial_{\mu_{i}}J\left(\mu \right)\right]=R_{\mu_{i}}\left[\Omega {\left[\mu \right]}_{i}\right]$$

which shows that the right-hand sides of (114) and (119) are equal. □

**Proof** **of** **Proposition** **9.** Starting with (115), we compute

(A113) $$J\left(\mu \right)=-\frac{1}{2}\langle \mu ,\Omega \left[\mu \right]\rangle =\frac{1}{2}\langle \mu ,\left(\mathrm{id}-\Omega -\mathrm{id}\right)\left[\mu \right]\rangle =\frac{1}{2}\left(\langle \mu ,L_{G}\left[\mu \right]\rangle -{∥\mu ∥}^{2}\right).$$

The quadratic form involving $L_{G}$ remains to be rewritten.

(A114) $$\begin{array}{cc} \langle \mu ,L_{G}\left[\mu \right]\rangle \; & =\sum_{i\in V}\langle \mu_{i},\mu_{i}-\Omega {\left[\mu \right]}_{i}\rangle =\sum_{i\in V}\left(∥\mu_{i}{∥}^{2}-\langle \mu_{i},\sum_{k\in N_{i}}\omega_{ik}\mu_{k}\rangle \right) \end{array}$$

(A115) $$\begin{array}{cc} \; & =\sum_{i\in V}\left(\underset{=1}{\underset{︸}{\sum_{k\in N_{i}}\omega_{ik}}}{∥\mu_{i}∥}^{2}-\sum_{k\in N_{i}}\omega_{ik}\langle \mu_{i},\mu_{k}\rangle \right)=\sum_{i\in V}\sum_{k\in N_{i}}\omega_{ik}\left(\langle \mu_{i},\mu_{i}-\mu_{k}\rangle \right) \end{array}$$

(A116) $$\begin{array}{cc} \; & =\frac{1}{2}\left(\sum_{i\in V}\sum_{k\in N_{i}}\omega_{ik}\left(\langle \mu_{i},\mu_{i}-\mu_{k}\rangle \right)+\sum_{k\in V}\sum_{i\in N_{k}}\omega_{ki}\left(\langle \mu_{k},\mu_{k}-\mu_{i}\rangle \right)\right), \end{array}$$

where the last sum results from the first one by interchanging indices *i* and *k*. Using the symmetry relations (110) and (111), we rewrite the second sum as

(A117) $$\begin{array}{cc} \langle \mu ,L_{G}\left[\mu \right]\rangle \; & =\frac{1}{2}\left(\sum_{i\in V}\sum_{k\in N_{i}}\omega_{ik}\left(\langle \mu_{i},\mu_{i}-\mu_{k}\rangle \right)+\sum_{i\in V}\sum_{k\in N_{i}}\omega_{ik}\left(\langle \mu_{k},\mu_{k}-\mu_{i}\rangle \right)\right) \end{array}$$

(A118) $$\begin{array}{cc} \; & =\frac{1}{2}\sum_{i\in V}\sum_{k\in N_{i}}\omega_{ik}\left(\langle \mu_{i},\mu_{i}-\mu_{k}\rangle +\langle \mu_{k},\mu_{k}-\mu_{i}\rangle \right) \end{array}$$

(A119) $$\begin{array}{cc} \; & =\frac{1}{2}\sum_{i\in V}\sum_{k\in N_{i}}\omega_{ik}{∥\mu_{i}-\mu_{k}∥}^{2} \end{array}$$

which completes the proof. □

**Proof** **of** **Lemma** **9.** We start with claim (b).

(b) Let $\mu \in D_{\Pi }$ and $X\in T_{\mu }D_{\Pi }$. Suppose that vector *X* is represented by a curve $\eta :\left(-\varepsilon ,\varepsilon \right)\to D_{\Pi }$ such that η(0)=μ and $\eta^{\prime }\left(0\right)=X$. In view of the definition (123) of $D_{\Pi }$, we have
  (A120) $$\eta \left(t\right)=\sum_{i\in [l]}\frac{p_{i}\left(t\right)}{\mathrm{tr}\pi_{i}}\pi_{i}\;\Rightarrow \;X=\sum_{i\in [l]}\frac{p_{i}^{\prime }\left(0\right)}{\mathrm{tr}\pi_{i}}\pi_{i}.$$
  Consequently, if $U=u_{1},...,u_{c}$ is a basis of $C^{c}$ that diagonalizes μ, then the tangent vector *X* is also diagonal in this basis U, and *X* commutes with μ, i.e., [μ,X]=0 and $X\in T_{\mu }^{c}D_{c}$. This proves (b).
(a) The bijection $D_{\Pi }\to S_{l}$ is explicitly given by
  (A121) $$\Phi_{\Pi }:D_{\Pi }\to S_{l},\;\sum_{i\in [l]}\frac{p_{i}}{\mathrm{tr}\pi_{i}}\pi_{i}\mapsto \left(p_{1},...,p_{l}\right).$$
  This is bijective according to the definition of $D_{\Pi }$. It remains to be shown that it is an isometry. Consider another tangent vector $Y\in T_{\mu }D_{\Pi }$. We know that μ,X,Y can all be diagonalized in a common eigenbasis. This basis is denoted again by U. Then, we can write
  (A122) $$\mu =\sum_{i\in [c]}{\tilde{p}}_{i}u_{i}u_{i}^{*},\;X=\sum_{i\in [c]}{\tilde{x}}_{i}u_{i}u_{i}^{*},\;Y=\sum_{i\in [c]}{\tilde{y}}_{i}u_{i}u_{i}^{*}$$
  and compute
  (A123) $$\begin{array}{cc} \iota^{*}g_{\mathrm{BKM},\mu }\left(X,Y\right)\; & =\int_{0}^{\infty }\mathrm{tr}\left(X{\left(\mu +\lambda I\right)}^{-1}Y{\left(\mu +\lambda I\right)}^{-1}\right)d\lambda \end{array}$$
  (A124) $$=\sum_{i\in [c]}\int_{0}^{\infty }\mathrm{tr}\left(\frac{{\tilde{x}}_{i}{\tilde{y}}_{i}}{{\left({\tilde{p}}_{i}+\lambda \right)}^{2}}u_{i}u_{i}^{*}\right)d\lambda$$
  (A125) $$=\sum_{i\in [c]}\frac{{\tilde{x}}_{i}{\tilde{y}}_{i}}{{\tilde{p}}_{i}}.$$
  Note that the vector $\tilde{p}=\left({\tilde{p}}_{1},...,{\tilde{p}}_{c}\right)$ comes from $\mu \in D_{\Pi }$. Therefore, the value $p_{j}/\mathrm{tr}\pi_{j}$ must occur $\mathrm{tr}\pi_{j}$ times in $\tilde{p}$ for every j∈[l]. This observation also holds for the vectors $\tilde{x}=\left({\tilde{x}}_{1},...,{\tilde{x}}_{c}\right)$ and $\tilde{y}=\left({\tilde{y}}_{1},...,{\tilde{y}}_{c}\right)$. Thus, the sum above can be reduced to
  (A126) $$\sum_{i\in [c]}\frac{{\tilde{x}}_{i}{\tilde{y}}_{i}}{{\tilde{p}}_{i}}=\sum_{j\in [l]}\frac{x_{j}y_{j}}{p_{j}},$$
  where $\left(p_{1},...,p_{j}\right)=\Phi \left(\mu \right)$, $\left(x_{1},...,x_{l}\right)=d\Phi \left[X\right]$ and $\left(y_{1},...,y_{l}\right)=d\Phi \left[Y\right]$. Taking into account that $\left(x_{1},...,x_{l}\right)$ and $\left(y_{1},...,y_{l}\right)$ are the images of X,Y under the differential dΦ, we conclude
  (A127) $$\iota^{*}g_{\mathrm{BKM},\mu }\left(X,Y\right)=\sum_{i\in [l]}\frac{x_{i}y_{i}}{p_{i}}\overset{\left(8\right)}{=}g_{\mathrm{FR},\Phi (\mu )}\left(d\Phi \left(X\right),d\Phi \left(Y\right)\right).$$
  This proves part (a).
(c) Part (c) is about the commutativity of the diagram.
  (A128) $$\begin{array}{cc} D_{\Pi }\left[r,hook,"\alpha_{\Pi }"\right]\left[d,"\Phi_{\Pi }"\right] & D_{\Pi_{U}}\left[d,"\Phi_{\Pi_{U}}"\right]\\ S_{l}\left[r,hook,"\beta_{\Pi }"\right] & S_{c}. \end{array}$$
  The horizontal arrows can be described as follows. Recall that $\Pi =\pi_{1},...,\pi_{l}$. $k_{i}=\mathrm{tr}\pi_{i}$ represents the dimensions of the images of the projectors $\pi_{i}$. For a fixed $p=\left(p_{1},...,p_{l}\right)\in S_{l}$, set
  (A129) $$P=\left(P_{1},...,P_{c}\right):=\left(\underset{k_{1}\;\mathrm{times}}{\underset{︸}{p_{1}/k_{1},...,p_{1}/k_{1}}},...,\underset{k_{l}\;\mathrm{times}}{\underset{︸}{p_{l}/k_{l},...,p_{l}/k_{l}}}\right)\in S_{c}.$$
  Then, $\alpha_{\Pi }$ is given by
  (A130) $$\alpha_{\Pi }\left(\sum_{i\in [l]}\frac{p_{i}}{k_{i}}\pi_{i}\right)=\sum_{j\in [c]}P_{j}u_{j}u_{j}^{*}\in D_{\Pi_{U}}\;\mathrm{and}\;\beta_{\Pi }\left(p_{1},...,p_{l}\right)=\left(P_{1},...,P_{c}\right)$$
  Diagram (A128) commutes according to the definition of the Φ maps.

□

**Proof** **of** **Lemma** **10.**

(i) Due to the commutativity of the components $\mu_{i}$ of μ∈Q, we can simplify the expression for the vector field of the QSAF as follows.
  (A131) $$\begin{array}{cc} R_{\mu }{\left[\Omega \left[\mu \right]\right]}_{i} & \overset{\left(106\right)}{=}R_{\mu_{i}}\left[\Omega {\left[\mu \right]}_{i}\right] \end{array}$$
  (A132) $$\begin{array}{cc} \; & \overset{\begin{array}{c} \left(64\right)\\ \left(28\right) \end{array}}{=}\sum_{k\in N_{i}}\omega_{ik}\left(\int_{0}^{1}\mu_{i}^{1-\lambda }\mu_{k}\mu_{i}^{\lambda }d\lambda -\mathrm{tr}(\mu_{i}\mu_{k})\mu_{i}\right) \end{array}$$
  (A133) $$\begin{array}{cc} \; & =\sum_{k\in N_{i}}\omega_{ik}\left(\mu_{i}\mu_{k}-\mathrm{tr}(\mu_{i}\mu_{k})\mu_{i}\right). \end{array}$$
  Define $\mu \in D_{\Pi ,c}$ such that all the components $\mu_{i}$ can be written as
  (A134) $$\mu_{i}=\sum_{r\in [l]}\frac{p_{r}^{i}}{\mathrm{tr}\pi_{r}}\pi_{r},\;p^{i}=\left(p_{1}^{i},...,p_{l}^{i}\right)\in S_{l},\;i\in V.$$
  Then, we can further simplify
  (A135) $$\mu_{i}\mu_{k}=\sum_{r\in [l]}\frac{p_{r}^{i}p_{r}^{k}}{{\left(\mathrm{tr}\pi_{r}\right)}^{2}}\pi_{r}\;\mathrm{and}\;\mathrm{tr}\left(\mu_{i}\mu_{k}\right)=\sum_{r\in [l]}\frac{p_{r}^{i}p_{r}^{k}}{\mathrm{tr}\pi_{r}}$$
  and, consequently,
  (A136) $$\begin{array}{cc} \sum_{k\in N_{i}}\omega_{ik}\left(\mu_{i}\mu_{k}-\left(\mu_{i}\mu_{k}\right)\mu_{i}\right)\; & =\sum_{r\in [l]}\sum_{k\in N_{i}}\omega_{ik}\left(\frac{p_{r}^{k}}{\mathrm{tr}\pi_{r}}-\left(\sum_{s\in [l]}\frac{p_{s}^{i}p_{s}^{k}}{\mathrm{tr}\pi_{s}}\right)\right)\frac{p_{r}^{i}}{\mathrm{tr}\pi_{r}}\pi_{r} \end{array}$$
  (A137) $$\begin{array}{cc} \; & =\sum_{r\in [l]}\frac{x_{r}}{\mathrm{tr}\pi_{r}}\pi_{r}, \end{array}$$
  where
  (A138) $$x_{r}:=\sum_{k\in N_{i}}\omega_{ik}\left(\frac{p_{r}^{k}}{\mathrm{tr}\pi_{r}}-\left(\sum_{s\in [l]}\frac{p_{s}^{i}p_{s}^{k}}{\mathrm{tr}\pi_{s}}\right)\right)p_{r}^{i}.$$
  Thus,
  $$R_{\mu }{\left[\Omega \left[\mu \right]\right]}_{i}=\sum_{r\in [l]}x_{r}/\left(\mathrm{tr}\pi_{r}\right)\pi_{r}.$$
  This has to be compared with the general form of a tangent vector( $X\in T_{\mu_{i}}D_{\Pi }$ given by (A120). The only condition the vector $p^{\prime }\left(0\right)$ in (A120) has to satisfy is that its components sum to 0. This also holds for $x=(x_{1},...x_{l})$. We conclude that $R_{\mu }{\left[\Omega \left[\mu \right]\right]}_{i}$ lies in $T_{\mu_{i}}D_{\Pi }$ for all i∈V or, equivalently, $R_{\mu }\left[\Omega \left[\mu \right]\right]\in T_{\mu }D_{\Pi ,c}$.
(ii) We write $\mu_{i}=U\mathrm{Diag}\left(S_{i}\right)U^{*}$ for all i∈V with $S_{i}\in S_{c}$ and express $R_{\mu }\left[\Omega \left[\mu \right]\right]$ in terms of S∈W as
  (A139) $$\begin{array}{cc} R_{\mu }{\left[\Omega \left[\mu \right]\right]}_{i} & =\sum_{k\in N_{i}}\omega_{ik}\left(\mu_{i}\mu_{k}-\mathrm{tr}(\mu_{i}\mu_{k})\mu_{i}\right) \end{array}$$
  (A140) $$\begin{array}{c} =U\mathrm{Diag}\left(\sum_{k\in N_{i}}\omega_{ik}\left(S_{i}\cdot S_{k}-\langle S_{i},S_{k}\rangle S_{i}\right)\right)U^{*} \end{array}$$
  (A141) $$\begin{array}{c} =U\mathrm{Diag}{\left(R_{S}\left[\Omega S\right]\right)}_{i}U^{*}. \end{array}$$

□

**Proof** **of** **Corollary** **3.** We write $D_{i}=U\mathrm{Diag}\left(\lambda_{i}\right)U^{*}$ for $\lambda_{i}\in R^{n}$ diagonalized in the *U* basis. Then, the initial condition for the QSAF S flow (109) is given by

(A142) $$\mu {\left(0\right)}_{i}=S{\left(1_{Q}\right)}_{i}\overset{\left(101\right)}{=}\Gamma \left(\sum_{k\in N_{i}}\omega_{ik}\left(-D_{k}\right)\right).$$

Then, set ${\tilde{D}}_{i}:=\sum_{k\in N_{i}}\omega_{ik}D_{k}=U\mathrm{Diag}\left({\tilde{\lambda }}_{i}\right)U^{*}$, where

(A143) $${\tilde{\lambda }}_{i}=\sum_{k\in N_{i}}\omega_{ik}\lambda_{k}\in R^{c}.$$

Recall further that Γ is computed in terms of the matrix exponential as specified by (66). Thus,

(A144) $$\mu {\left(0\right)}_{i}=\Gamma \left(-{\tilde{D}}_{i}\right)=\frac{\exp_{m}\left(-{\tilde{D}}_{i}\right)}{\mathrm{tr}\exp_{m}\left(-{\tilde{D}}_{i}\right)}=\frac{U\exp_{m}\left(-\mathrm{Diag}\left({\tilde{\lambda }}_{i}\right)\right)U^{*}}{\mathrm{tr}(U\exp_{m}\left(-\mathrm{Diag}\left({\tilde{\lambda }}_{i}\right)\right)U^{*})}=U\frac{\mathrm{Diag}(\exp\left(-{\tilde{\lambda }}_{i}\right))}{\mathrm{tr}\exp_{m}\left(-\mathrm{Diag}\left({\tilde{\lambda }}_{i}\right)\right)}U^{*}.$$

This shows that all the $\mu {\left(0\right)}_{i}^{\prime }s$ are diagonalized by the same basis U and $\mu \left(0\right)\in D_{\Pi_{U},c}$, and we can apply Proposition 10 (ii). Therefore, the vector field of the quantum state assignment S flow is also diagonalized in the U basis, and we solve simply for the diagonal components. The quantum S-flow equation can be written as

(A145) $${\dot{\mu }}_{i}=U\mathrm{Diag}\left(R_{S_{i}}\left[\Omega S\right]\right)U^{*},\;\mu {\left(0\right)}_{i}=U\mathrm{Diag}\left(S\left(1_{W}\right)\right)U^{*}$$

with the classical similarity map *S* defined in terms of the data vectors $\lambda_{i}$ and $\mu_{i}$ related to $S_{i}\in S_{c}$ by $\mu_{i}=U\mathrm{diag}\left(S_{i}\right)U^{*}$. The solution to this system is

(A146) $$\mu_{i}\left(t\right)=U\mathrm{Diag}\left(S_{i}\left(t\right)\right)U^{*},$$

where S∈W solves the classical S-flow equation $\dot{S}=R_{S}\left[\Omega S\right]$ and $S\left(0\right)=S\left(1_{W}\right)$. □
